# Cell-Type Specific DNA Methylation Patterns Define Human Breast Cellular Identity

**DOI:** 10.1371/journal.pone.0052299

**Published:** 2012-12-20

**Authors:** Petr Novak, Martha R. Stampfer, Jose L. Munoz-Rodriguez, James C. Garbe, Mathias Ehrich, Bernard W. Futscher, Taylor J. Jensen

**Affiliations:** 1 Arizona Cancer Center, The University of Arizona, Tucson, Arizona, United States of America; 2 Department of Pharmacology and Toxicology, College of Pharmacy, The University of Arizona, Tucson, Arizona, United States of America; 3 Life Sciences Division, Lawrence Berkeley National Laboratory, Berkeley, California, United States of America; 4 Biology Centre ASCR, v.v.i., Institute of Plant Molecular Biology, Ceske Budejovice, Czech Republic; 5 Sequenom, Inc., San Diego, California, United States of America; 6 Sequenom Center for Molecular Medicine, John Hopkins Ct, San Diego, California, United States of America; University of North Carolina School of Medicine, United States of America

## Abstract

DNA methylation plays a role in a variety of biological processes including embryonic development, imprinting, X-chromosome inactivation, and stem cell differentiation. Tissue specific differential methylation has also been well characterized. We sought to extend these studies to create a map of differential DNA methylation between different cell types derived from a single tissue. Using three pairs of isogenic human mammary epithelial and fibroblast cells, promoter region DNA methylation was characterized using MeDIP coupled to microarray analysis. Comparison of DNA methylation between these cell types revealed nearly three thousand cell-type specific differentially methylated regions (ctDMRs). MassARRAY was performed upon 87 ctDMRs to confirm and quantify differential DNA methylation. Each of the examined regions exhibited statistically significant differences ranging from 10–70%. Gene ontology analysis revealed the overrepresentation of many transcription factors involved in developmental processes. Additionally, we have shown that ctDMRs are associated with histone related epigenetic marks and are often aberrantly methylated in breast cancer. Overall, our data suggest that there are thousands of ctDMRs which consistently exhibit differential DNA methylation and may underlie cell type specificity in human breast tissue. In addition, we describe the pathways affected by these differences and provide insight into the molecular mechanisms and physiological overlap between normal cellular differentiation and breast carcinogenesis.

## Introduction

DNA methylation is an epigenetic mark located on the carbon-5 position of cytosine residues in mammalian genomes, primarily on the cytosine within a cytosine-guanine sequence in differentiated cells [1]. This epigenetic modification of genomic DNA plays a role in a variety of biological processes including embryonic development, imprinting, and X-chromosome inactivation [2]–[4]. In addition, the differentiation of pluripotent stem cells to comprise the various tissues and cell types within the body is thought to be controlled by epigenetic mechanisms including DNA methylation [5]. Upon terminal differentiation, these epigenomic changes become fixed and contribute to the maintenance of cellular identity and function and are thus critical for normal tissue function and architecture [4], [6].

In addition to its role in normal physiology, aberrant DNA methylation has been shown to contribute to numerous disease states including various types of cancer [7]–[11]. In general, the methylome of a cancer cell tends to contain two distinct epigenetic phenomena: global hypomethylation and regional hypermethylation. Global hypomethylation occurs primarily within repetitive DNA sequences and pericentromeric regions that exhibit high levels of DNA methylation in normal cells [12], [13]. Conversely, regions often unmethylated in normal cells, such as CpG islands and gene promoter regions, typically become hypermethylated during carcinogenesis in a non-random manner [14]–[16]. In the case of breast cancer, aberrant DNA methylation is a known contributor to the disease [17]–[19]; however, the molecular mechanisms associated with disease development and progression are still not well understood.

Differential DNA methylation patterns between distinct normal tissues and between normal and cancerous tissue are well established [1], [7]–[11], [16], [20]–[23]. Previous studies have defined hundreds of tissue- and cancer- specific differentially methylated regions (tDMRs, cDMRs) when comparing multiple human tissues [8], [11], [16], [21], [22], [24]–[26]; however, DNA methylation patterns among distinct differentiated cell types from a single non-cancerous tissue are less characterized. To address this, we examined the DNA methylation patterns of two distinct differentiated cell types within one organ system: human mammary epithelial cells (HMEC) and human mammary fibroblasts (HMF). These cell strain pairs were derived from the same normal, non-cancerous breast tissue and have been shown to be differentially methylated in regions surrounding miRNAs [27], [28]. Overall, the extension of previous work to identify differentially methylated gene promoters may help further understanding of normal human breast tissue function as well as aberrations that occur in maladies associated with these cell types.

We performed methylcytosine immunoprecipitation (MeDIP) coupled to Affymetrix human promoter microarrays to assess the cell-type specific methylation patterns between isogenic pairs of HMEC and HMF. The data suggest that there are more than 3000 cell type specific differentially methylated regions (ctDMRs) when comparing these samples. The functional categorization of affected promoters using gene ontology (GO) testing revealed the enrichment of many categories of genes important in developmental processes, consistent with a potential contribution to cell lineage maintenance. Comparison of the identified ctDMRs also linked those regions methylated in fibroblasts to those hypermethylated in breast cancer, while those methylated in HMEC were linked to regions hypomethylated in breast cancer.

## Materials and Methods

### Cell Culture

Finite lifespan pre-stasis human mammary epithelial cells (HMEC) from specimens 184 (batch D), 48R (batch T), and 240L (batch B), were derived from reduction mammoplasty tissue of women aged 21, 16, and 19 respectively. Cells were initiated as organoids in primary culture in serum-containing M85 medium supplemented with 0.1 nM oxytocin (Bachem) and maintained in M87A medium supplemented with oxytocin and 0.5 ng/mL cholera toxin [29]. HMF were separated from the epithelial cells during processing of the surgical discard tissue and grown in pure culture as previously described [30], [31]. Fibroblasts from specimens 184, 48, and 240L were obtained from the same reduction mammoplasty tissue and were grown in DMEM/F12 media containing 10% FBS and 10 µg/ml insulin and further propagated in DMEM/F12 with 10% FBS [29]. DNA was isolated from samples during the following passage numbers: HMEC 184 p5; HMEC 48 p4; HMEC 240 p5; HMF 184 p9; HMF 48 p7; HMF 240 p3. HMEC at these low passage numbers include a mixture of luminal, myoepithelial, and progenitor cells [29], [32]. Breast cancer cell lines BT549, UACC-1179, UACC-3199, HS578T were cultured as previously described [22]. Genomic DNA from MDA-MB-231, T47-D, and MCF7 was purchased from the American Type Culture Collection (ATCC; Manassas, VA).

### Nucleic Acid Isolation

Genomic DNA from cultured cells was isolated using the DNeasy Blood and Tissue Kit as described by manufacturer (Qiagen; Valencia, CA). The quantity and relative quality of each sample was assessed using absorbance at 260 nm using the NanoDrop 1000 Spectrophotometer (NanoDrop; Wilmington, DE).

### DNA Methylation MicroarrayAnalysis

MeDIP was performed as previously described [33], [34]. The immunoprecipitated DNA fraction (200 ng) from HMEC and HMF from each genotype was processed and hybridized to an Affymetrix GeneChip 1.0R Human Promoter Array (Affymetrix; Santa Clara, CA) according to manufacturer’s protocol.

### Microarray Data Analysis

Raw microarray data (CEL files) were processed and analyzed in an R programming environment using the affy, affxparser, preprocessCore,spatstat, and ACME packages. Briefly, hybridization signal was log base 2 transformed. On each array, spatial normalization was performed to remove any potential local bias in hybridization signal. Next, the microarray signal was quantile normalized.

To find differentially methylated regions, signals from three genotype in epithelial samples and fibroblast were compared using paired t-test for each probe on the array. The resultant profile of t-statistics along chromosomes was then analyzed using the Bioconductor ACME package using a sliding window of 300 base pairs to detect regions where the t-statistic deviated positively or negatively from zero signifying methylation in HMEC or HMF, respectively. To correct for multiple testing, we have performed the same analysis upon data where the position of the probes was randomly selected. This analysis was based on the premise that any region detected as positive upon randomly selected data is a false positive. This analysis was then used to calculate a threshold from which allows the detection of differentially methylated regions with a false positive rate below 0.05.

All data analysis was done using the NCBI36/hg18 build of the human genome assembly obtained from the UCSC genome browser (http://genome.ucsc.edu). For each positive region, the refseq transcript with the closest transcription start site (TSS) was identified. Similarly, the closest CpG islands relative to each ctDMRwere identified using the CpG Island Track from UCSC. We have also used publicly available ChIP-Seq data from post-stasis HMEC that was deposited into the UCSC Genome Browser to assess the histone modification profile relative to the identified ctDMRs [35], [36]. For these analyses, ctDMRs which were methylated in HMEC and HMF were analyzed separately and enrichment signal from the downloaded UCSC track was extracted for each ctDMR for a distance range of 10 kbp. For visualization purposes, a moving average was calculated to show the average histone modification profile across each region. To check for array coverage bias, we reproduced all analysis with randomly positioned regions.

### Gene Ontology (GO) Analysis

Each ctDMR located within 2 kbp of the transcription start site of a gene and for which an Entrez ID was available was included in this analysis. As a control set of genes, we used all genes with an Entrez ID that were covered by the microarray. Significantly enriched GO terms were identified using the topGO package using the *elim* algorithm and a *fisher* exact test to evaluate gene counts [37].

Microarray data are available in ArrayExpress database (accession E-MEXP-3651, www.ebi.ac.uk/arrayexpress/)

### EpiTYPER Analysis

Primers were designed to regions of interest using EpiDesigner software (http://epidesigner.com). Genomic DNA sequences were obtained from the UCSC genome browser (http://genome.ucsc.edu; hg18 build, March 2006) and loaded in to EpiDesigner. Primer sequences were exported from EpiDesigner and primers were ordered from Integrated DNA Technologies (Coralville, IA) and were received after standard desalting at a concentration of 100 µM.

Genomic DNA was subjected to sodium bisulfite conversion using the Zymo EZ DNA Methylation Kit (Zymo, Orange, CA). Genomic DNA (1.2 µg) was added to HPLC grade water and 5 µL of M-Dilution Buffer (Zymo) in 50 µL total volume and incubated at 37°C for 15 minutes. Upon completion, 100 µL of prepared conversion reagent (Zymo) was added to each sample and samples were incubated for 21 cycles of 95°C for 30 seconds followed by 50°C for 15 minutes. After cycling incubation, sodium bisulfite converted DNA samples were purified using a column based method (Zymo) according to the manufacturer’s instructions and eluted in 130 µL of HPLC grade water for use in subsequent PCR reactions.

Sodium bisulfite converted DNA (1 µL) was added to HPLC grade water (1.36 µL), 10X PCR Buffer (0.5 µL; Sequenom, San Diego, CA), dNTPs (0.04 µL; 200 µM; Sequenom), FastStart PCR enzyme (0.1 µL; 0.5U; Sequenom) and region specific PCR primers (2 µL; 200 nM each). Samples were then subjected to brief centrifugation and incubated according to the following cycling parameters: 94°C for 4 minutes; 45 cycles of 94°C for 20 seconds, 56°C for 30 seconds, 72°C for 1 minute; 72°C for 3 minutes.

A shrimp alkaline phosphatase (SAP) mixture was prepared by adding 0.3 µL (0.3U) SAP (Sequenom) to 1.7 µL HPLC grade water for each reaction. Upon completion of the PCR reaction, 2 µL of the SAP mixture was added to each reaction and the resultant mixtures were then incubated at 37°C for 20 minutes followed by 85°C for 5 minutes to inactivate the SAP enzyme. A portion of each sample (1 µL) was then transferred to a new 384-well PCR plate containing HPLC water (1.236 µL), 5X T7 Polymerase Buffer (0.38 µL; 0.64X; Sequenom), T-Cleavage Mix (0.094 µL; Sequenom), DTT (0.094 µL; 3.14 mM; Sequenom), T7 RNA/DNA polymerase (0.17 µL; Sequenom), and RNase A (0.026 µL; 0.09 mg/ml; Sequenom) and incubated at 37°C for 3 hours. Upon completion of in vitro transcription and base-specific cleavage, HPLC grade water (15 µL) and 6 mg clean desalting resin (Sequenom) were added to each reaction. Samples were rotated for 5–10 minutes at room temperature and a portion of each reaction (∼15 nL) was then spotted on to SpectroCHIP II chips (Sequenom) and analyzed using a MassARRAY Analyzer 4 MALDI-TOF mass spectrometer (Sequenom).

Data were initially viewed, spectra quality checked, and methylation values collected using EpiTYPER software (Build 1,0,5,77). Methylation values were exported from EpiTYPER and analysis was performed in an R programming environment. Poor quality data were removed prior to further analysis.

## Results

Human reduction mammoplasty tissue from each of three females was processed to separate HMEC from HMF. Each subpopulation was cultured and DNA harvested prior to reaching confluency. Alterations in DNA methylation have been demonstrated to occur in HMEC that have overcome a first defined HMEC stress-associated senescence barrier (stasis); therefore, only HMEC able of responding to stress through the upregulation of p16 and thus defined as pre-stasis were used for these studies [26], [29], [38]. DNA methylation patterns were assessed in three isogenic pairs of HMEC and HMF using MeDIP coupled to GeneChip Human Promoter 1.0R Arrays. This array covers the promoter regions, defined as 7.5 kb upstream through 2.45 kb downstream of 5' transcription start sites, of approximately 25,000 human genes. Analysis of the three isogenic cell strain pairs revealed a total of 2808 ctDMRs of which 1236 were methylated in HMF and 1572 were methylated in HMEC (Table 1; Table S1). Identified ctDMRs correspond to approximately 2.4% of analyzed genome (i.e. 97.6% of regions are without a significant methylation change). Taken together, these microarray data describe the presence and genomic location of thousands of ctDMRs in human breast tissue.

**Table 1:** Number of DMR.

**Tissue specific methylation**		
Methylated in epithelium		1572
Methylated in fibroblasts		1236
total:		2808
**Cancer specific methylation**		
Hypomethylated	1473	
Hypermethylated	2033	
total:	3506	

**Figure 1:**
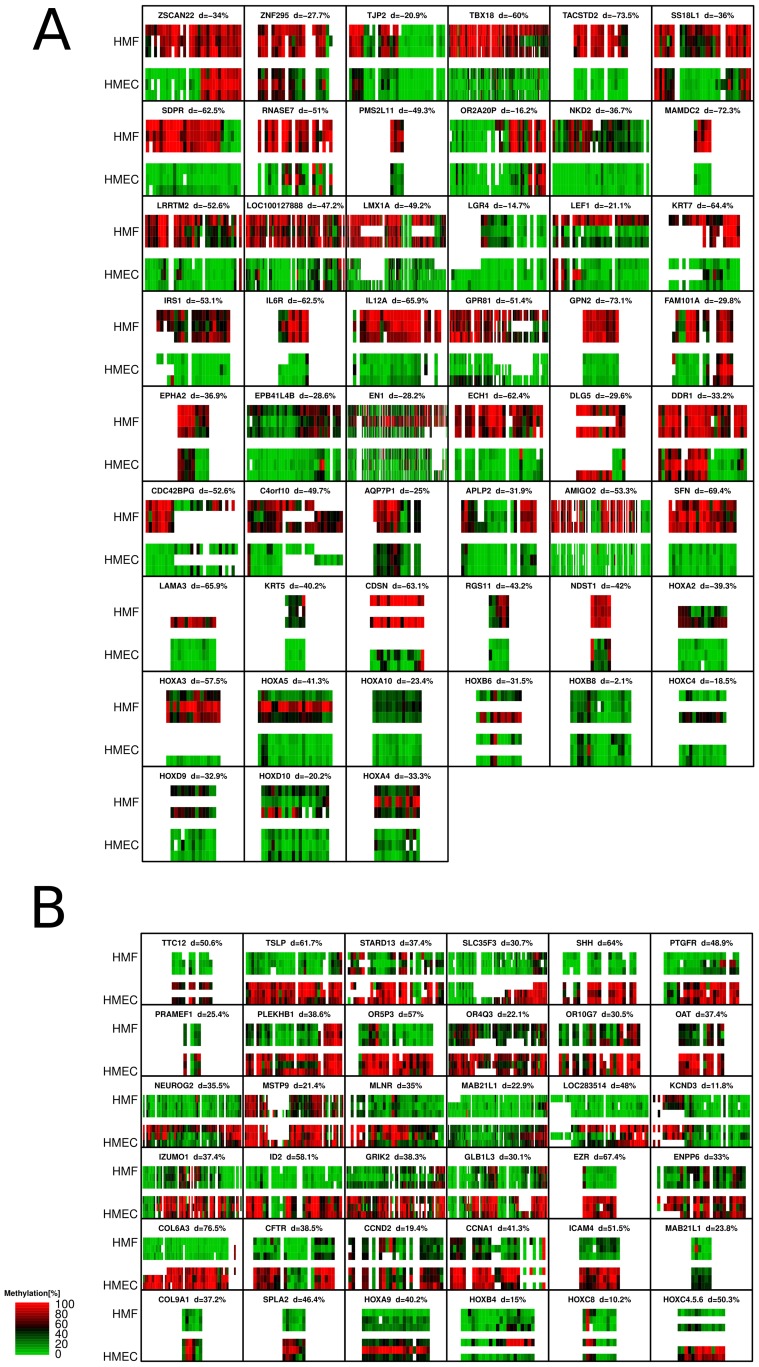
Validation of microarray results using MALDI-TOF mass spectrometry (MassARRAY). CtDMRs are labeled by the gene symbol of the closest gene. Each ctDMR is shown as a heatmap; methylation level for each informative CpG fragment is shown in columns and analyzed samples are organized in rows. Each colored rectangle corresponds to 1 CpG unit. All three genotypes were analyzed. White spaces are shown when the quality of data was not sufficient to assess methylation. **A**) ctDMRs which are methylated in HMF according to microarray. **B)** ctDMRs which are methylated in HMEC according to microarray. A more detailed view for each analyzed region is included in Information S1.

Confirmatory quantitative mass spectrometry (MassARRAY) was performed on each of the three paired cell strains across 87 ctDMRs, 36 which were hypermethylated in HMEC and 51 hypermethylated in HMF. DMRs were selected for validation to both validate the microarray results and, for a subset of the regions, to provide additional information about regions previously identified as aberrantly methylated in breast cancer. Using this method, the minimal detected mean difference in methylation levels was 10% and reached as high as 60–70% in some regions (Figures 1A and 1B). Each of these differences was statistically significant (p<0.05; Wilcox Test; Information S1). Overall, these data confirm the differential methylation observed using microarray analysis, suggesting the identification of ctDMRs was robust and reproducible. Additionally, MassARRAY analysis also revealed subtle interindividual variability in DNA methylation in a portion of analyzed loci. For example, while still showing cell type differential methylation, LEF1 also exhibits interindividual differences in DNA methylation within each distinct cell type.

**Figure 2:**
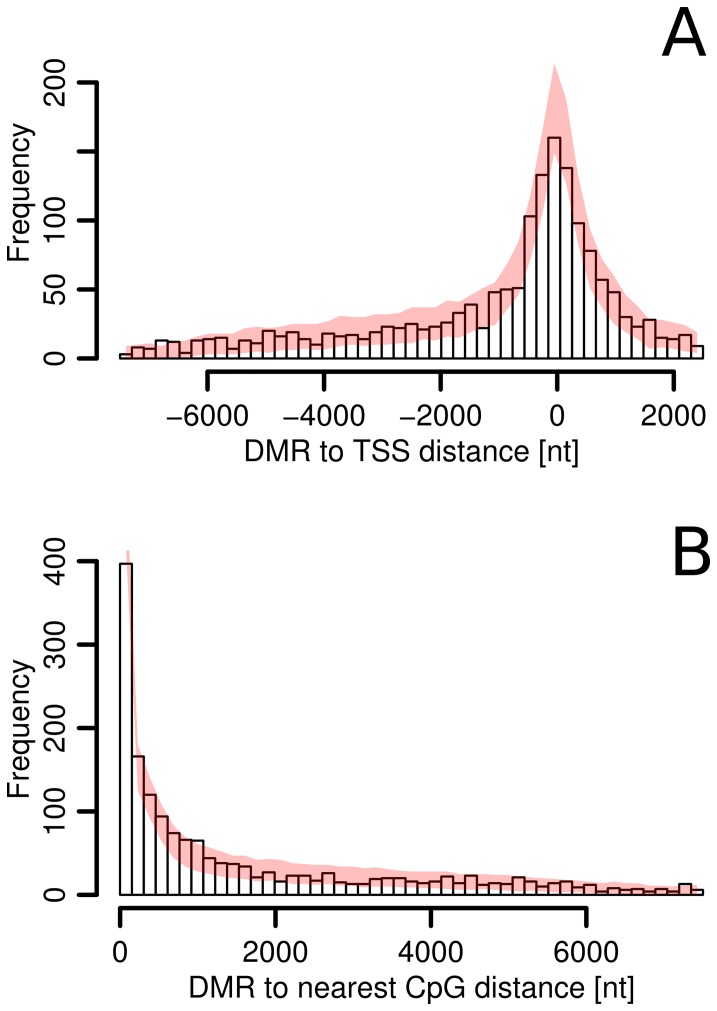
Distribution of ctDMRs. A) Position of ctDMR relative to TSS. Negative and positive distances correspond to sequences upstream and downstream to TSS, respectively. **B)** Position of ctDMR relative to the nearest CpG island. For both charts, pink lines show the expected distribution if DMRs have random localization and uneven microarray coverage is taken into consideration. 99% confidence interval based on the simulation is shown.

We next wanted to examine whether ctDMRs coincide with specific regions within promoters. For each identified ctDMR, we mapped the closest transcription start site (TSS) and the associated genes. The majority of the detected ctDMRs were located within1500 bp relative to the nearest TSS (Figure 2A). To evaluate whether this pattern was indicative of a significant portion of ctDMRs being localized near TSS, we examined the coverage of the microarray probes in each region and found that the increase in ctDMR frequency near TSS was mirrored by an increase in microarray coverage in these areas suggesting that the distribution of ctDMRs within promoters is not region specific. The distance from each identified ctDMR to the nearest CpG island was also calculated to characterize the coincidence of ctDMRs and CpG islands. Although the majority of ctDMRs were located within regions adjacent to CpG islands (CpG island shores) consistent with previous reports [25], this pattern did not deviate from what would be expected based upon microarray coverage (Figure 2B). Overall, these data suggest that the genomic distribution of ctDMRs within promoter regions is not directly linked to either TSS or CpG island position.

**Figure 3:**
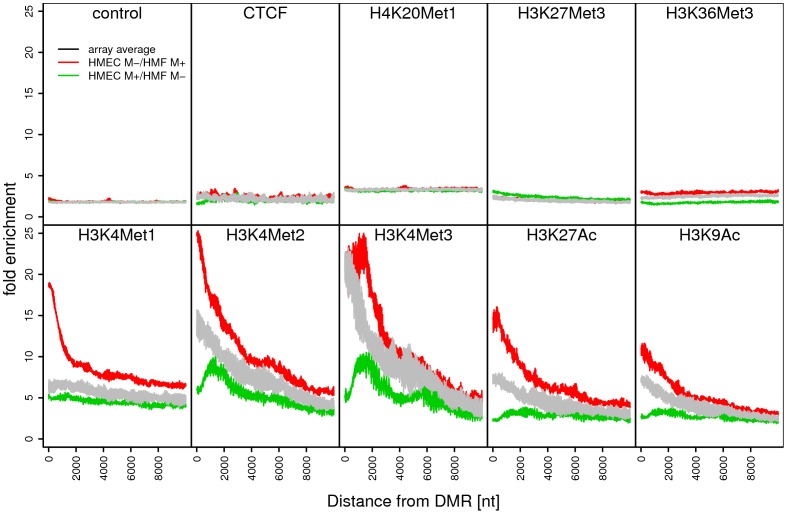
Average histone modifications in post-stasis post-selection HMEC in regions proximal to ctDMRs. For each group of ctDMRs, the signal enrichment from ChIP-Seq experiment performed on post-stasis HMEC was analyzed [35], [36]. All data points were grouped and a moving average along the distance from each ctDMR is shown. The microarray average was calculated from the same number of regions as ctDMR but randomly placed in the genome according the array coverage. Control profile is derived from input DNA signal of a chip-seq experiment. M+ = hypermethylation; M- = hypomethylation.

Since DNA methylation has previously been linked to other layers of epigenetic regulation, we have compared our results with previously published histone modification data. Histone modification data on normal pre-stasis HMEC were not available; the closest data available on finite lifespan HMEC was obtained from Ernst et al [35], [36]. The commercially available HMEC used in that publication (Lonza-2551) are post-stasis HMEC that have overcome the stasis senescence barrier, associated with silencing of the p16^INK4A^ promoter [38]–[40]. This type of post-stasis HMEC (referred to as post-selection or vHMEC [38], [41], [42]) is known to have incurred numerous changes in gene expression, lineage markers, and epigenetic marks compared to normal pre-stasis HMEC [26], [29], [40], [41], [43]. However, for the purposes of this study, the histone modification profiles of post-stasis HMEC were assessed within each of the identified ctDMRs (Figure 3). CtDMRs with reduced levels of DNA methylation in pre-stasis HMEC relative to HMF exhibited an association with the permissive histone marks H3K36Met3, H3K4Met1, H3K4Met2, H3K4Met3, H3K9Ac, and H3K27Ac found in the post-stasis HMEC. Conversely, regions hypermethylated in pre-stasis HMEC relative to HMF were not associated with the aforementioned permissive modifications but rather showed a weak association with the repressive H3K27Met3 modification in post-stasis HMEC. Regions of differential DNA methylation were not associated with CTCF or H4K20Met1. Taken together, these data reinforce the frequent overlap of multiple layers of epigenetic regulation within a genomic locus.

To explore a potential functional role of individual ctDMRs, we performed Gene Ontology (GO) classification analysis. All genes for which the TSS was within 2 kb of a ctDMR were used in this analysis. GO classification revealed multiple categories of genes that are frequently associated with ctDMRs (Table S2). We observed a significant overrepresentation of ctDMRs associated with genes involved in numerous developmental processes. These groups contained many classical developmental regulators including members of the HOX and SOX families of transcription factors. In addition, ctDMRs were associated with biological processes involved in cell-cell adhesion, a group that includes members of the ITG and PCDH gene families. Genes whose molecular function is based upon transcription factor activity and sequence-specific DNA binding were overrepresented, as were genes located in the extracellular region of the cell.

**Figure 4:**
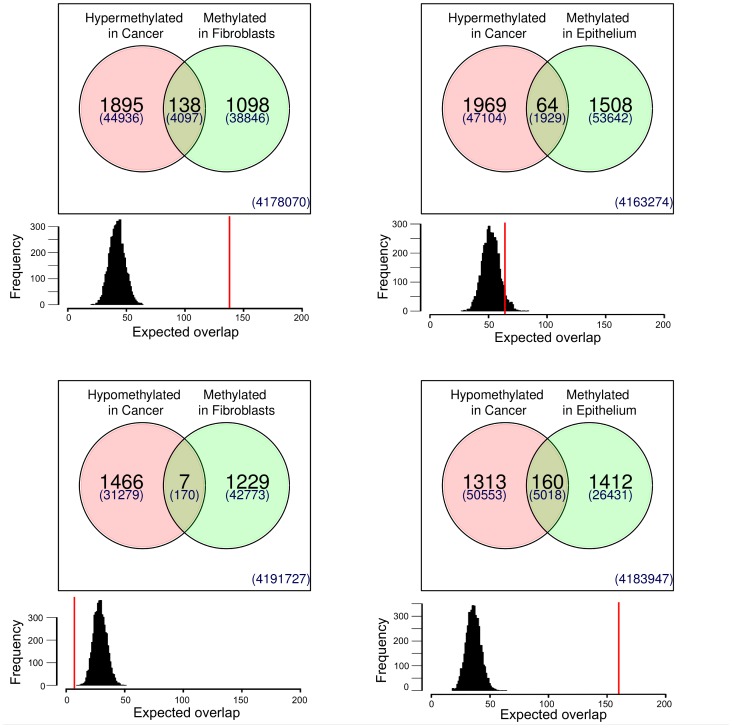
Comparison of ctDMRs with breast cancer specific DMRs. Number of unique and common DMRs is shown in Venn diagrams. Data for breast cancer specific DMR were reported previously [23]. Numbers in Venn diagrams show the number of regions; number of probes on the microarrays which are covering these region are show as numbers in parenthesis. Histograms below Venn diagrams show expected number of overlapping regions if positions of DMRs are random. Histogram is based on 5000 iterations of simulation. Red line shows the observed number of overlapping DMR.

**Figure 5:**
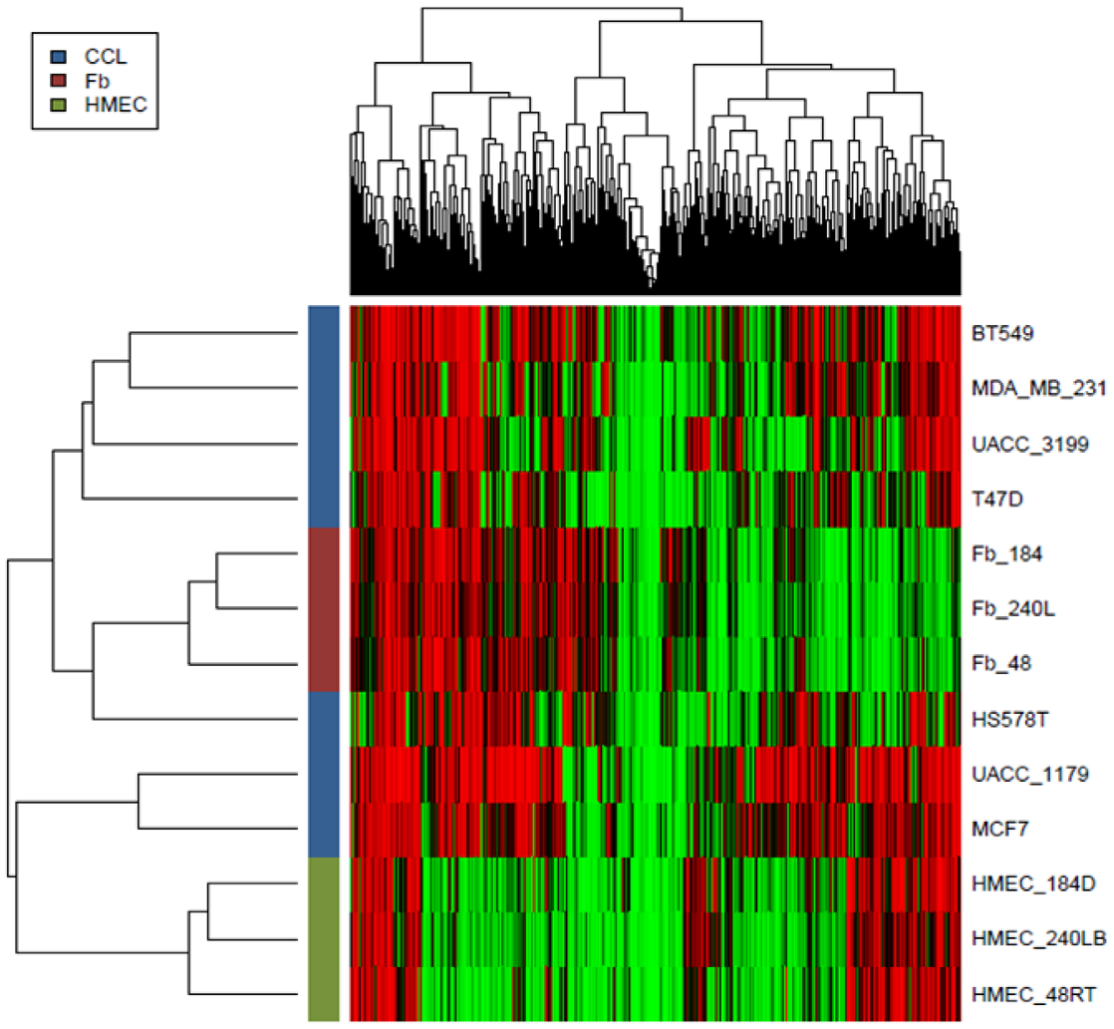
Comparison of methylation status in ctDMRs in HMF (Fb), HMEC and cancer cell lines (CCL) Methylation status of CpG sites 87 ctDMRs was analyzed by MassARRAY. Individual CpG and sample types were ordered by hierarchical clustering. Within heatmap, red and green correspond to hypermethylation and hypomethylation respectively.

Finally, we sought to determine if the ctDMRs uncovered in this study overlapped with previously identified aberrantly methylated regions in breast cancer [23]. Since the same MeDIP coupled to the same microarray platform was used in this previous study to identify cDMRs, the identified ctDMRs could be directly compared to the previously described cDMRs. Interestingly, the number of aberrant tumor-specific DMRs identified in breast cancer was only 25% higher than the number of identified tissue specific DMRs (Table 1). Comparison of the two DMR types reveals a high level of coincidence in their location (Figure 4). Simulations were performed to determine if the level of co-occurrence could be explained by chance. On average, regions hypermethylated in HMF were also hypermethylated in breast cancer when both were compared to pre-stasis HMEC. Conversely, regions which exhibited higher levels of methylation in pre-stasis HMEC (and thus were hypomethylated in HMF) showed a hypomethylation pattern in breast cancer relative to HMEC. This pattern was further confirmed through MassARRAY analysis of 87 ctDMRs in breast cancer cell lines (Figure 5). In the majority of cases, the methylation pattern of breast cancer cell lines more closely resembles that of HMF rather than HMEC. Taken together, these data indicate a significant overlap in the DNA methylation patterns between HMF and breast cancer.

## Discussion

We performed MeDIP coupled to human promoter microarrays upon three isogenic cell strain pairs of HMEC and HMF to identify regions that show distinct DNA methylation patterns when comparing these two differentiated cell types. These data identify thousands of regions which show statistically significant differences in DNA methylation. The identified ctDMRs are often associated with genes involved in development and differentiation and these genes often act as transcription factors implicated in cell patterning. Finally, ctDMRs were linked to regions aberrantly methylated during breast carcinogenesis, suggesting that ctDMRs are not only important in the maintenance of a differentiated state, but also in carcinogenesis. Overall, we provide an epigenomic map detailing DNA methylation within gene promoter regions in two distinct cell types derived from a single human mammary gland.

Numerous previous studies have identified tissue specific DNA methylation; however, many of these studies compare different tissue types derived from different individuals [20]–[22]. We sought to eliminate these potential contributors to differential methylation by utilizing cell strains derived from the same tissue from three individuals. This is important because our MassARRAY analysis suggests the presence of small but detectable inter-individual variability in cell-type specific DNA methylation. Previous studies were conducted comparing DNA methylation levels within different cell types within human mammary tissue; however, these studies compared adult tissue stem cells to differentiated luminal epithelial cells [44]. This was outside the scope of our project since we focused on identifying the differences between two differentiated cell lineages; however, both studies provide data that may be critical to understanding both normal breast function and breast carcinogenesis.

The comparison of DNA methylation patterns between HMEC and HMF uncovered the concordant differential methylation of numerous contiguous gene families (HOXA, HOXB, PCDH, OR; Table S2), suggesting that the long range epigenetic silencing of gene family clusters observed in many types of cancer, including mammary neoplasia, may have co-opted a pre-existent developmental mechanism to regulate large chromosomal regions [16], [23], [45]–[48]. This is further supported by the fact that many of the agglomerates of ctDMRs, including members of the HOXA, PCDH, and OR gene family clusters, are also targeted for aberrant long range epigenetic silencing in breast cancer [16], [23]. While the data suggest an overlap between ctDMRs and aberrantly methylated regions in cancer, further studies are needed to understand the mechanism by which these are extended to large genomic regions.

The search for biomarkers associated with cancer development and progression is an area of active research. It is of particular interest to discover and utilize biomarkers which can be obtained by non-invasive methods [49]. Since nucleic acids from tumors are present in the blood of an affected person, they are an attractive entity by which cancer diagnostics may be based. One way tumor nucleic acids are detected and differentiated from the abundance of nucleic acids present from other tissues is through the use of DNA methylation patterns. These data suggest that many of the regions which are differentially methylated in a cell-type specific manner are also the targets of aberrant DNA methylation in cancer. This finding should be considered in clinical utilization of such regions since, for example, a region may be methylated in either a breast tumor or an apoptotic HMF, potentially resulting in a false positive diagnosis. Particular attention should thus be paid to the selection of ctDMRs as biomarkers for non-invasive cancer detection.

The overlap of regions identified as aberrantly methylated in breast cancer were compared to those identified in this study as ctDMRs. This comparison suggests that regions hypermethylated in HMF are also hypermethylated in breast cancer samples and those regions identified to be hypomethylated in cancer were hypomethylated in HMF. Interestingly, hierarchical clustering of HMEC, HMF, and cancer cell lines differentiates the cancer cell lines into two groups: a smaller group which has a DNA methylation pattern more resembling HMEC and a larger group which has a DNA methylation pattern more resembling HMF (Figure 5). While the numbers are small, previous studies have shown that tumor cell lines which cluster with HMF (BT549, MDA-MB-231, T47D, and HS578T) all have *in vitro* invasive potential while MCF7, which clusters with HMEC, does not [50]. It is important to note that breast cancer subtype may also contribute to this clustering since many of those samples which cluster with HMF also have been classified as basal-like while MCF7 is of the luminal subtype. These data suggest that those differences in DNA methylation that differentiate epithelial from fibroblast cells in the breast may be associated with those that underlie breast cancer metastasis. Specifically, the identified overlap between regions methylated in HMF and those aberrantly methylated in breast cancer indicates that transition of a normal HMEC through the process of tumorigenesis can mimic the process of epithelial to mesenchymal transition (EMT). While hijacking mechanisms common to normal developmental processes during carcinogenesis has been suggested previously [21], [22], [24], [25], this study using isogenic cell strain pairs has shown strong statistical evidence for this process. Identification of ctDMRs, like those described herein, may provide a pool of genes which are targets of aberrant methylation during malignant transformation.

While we have identified thousands of ctDMRs within the human mammary gland, it is likely that there are thousands more. Our data were inherently biased through the use of a human promoter microarray to focus only upon regions surrounding gene TSS. Even though it was suggested previously that location DMR is biased toward TSS and CpG islands, we were not able to confirm such bias with our experiment [20], [24]. Our data suggest that ctDMRs are distributed in non-CpG and CpG regions with the same frequency. Additionally, recent studies using MeDIP-Seq or MethylC-Seq have concluded that many regions of differential methylation are not limited to promoter but are also located within intergenic or intragenic regions, something that our study does not address [1], [5], [25]. Even with these limitations these data suggest that there are thousands of ctDMRs which consistently exhibit differential DNA methylation and may underlie cell type specificity in human breast, describe the pathways affected by these differences, and provide insight into the molecular mechanisms and physiological overlap between normal cellular differentiation and breast carcinogenesis.

## Supporting Information

Table S1 List of all detected ctDMRs, coordinates of regions correspond to NCBI36/hg18 build of the human genome assembly.
(XLS)

Table S2 **Significantly enriched Gene Ontology terms when set of genes in proximity of each ctDMR were analyzed.**
(XLSX)

Information S1 **Detailed view of validated ctDMR.**
(PDF)
