# Supplementary material for: Cell-Type Specific DNA Methylation Patterns Define Human Breast Cellular Identity
Source: PLoS One. 2012 Dec 20;7(12):e52299. doi: 10.1371/journal.pone.0052299 (PMC3527522; doi:10.1371/journal.pone.0052299)
Supplement: Information S1 — Detailed view of validated ctDMR. (PDF) [file pone.0052299.s003.pdf]

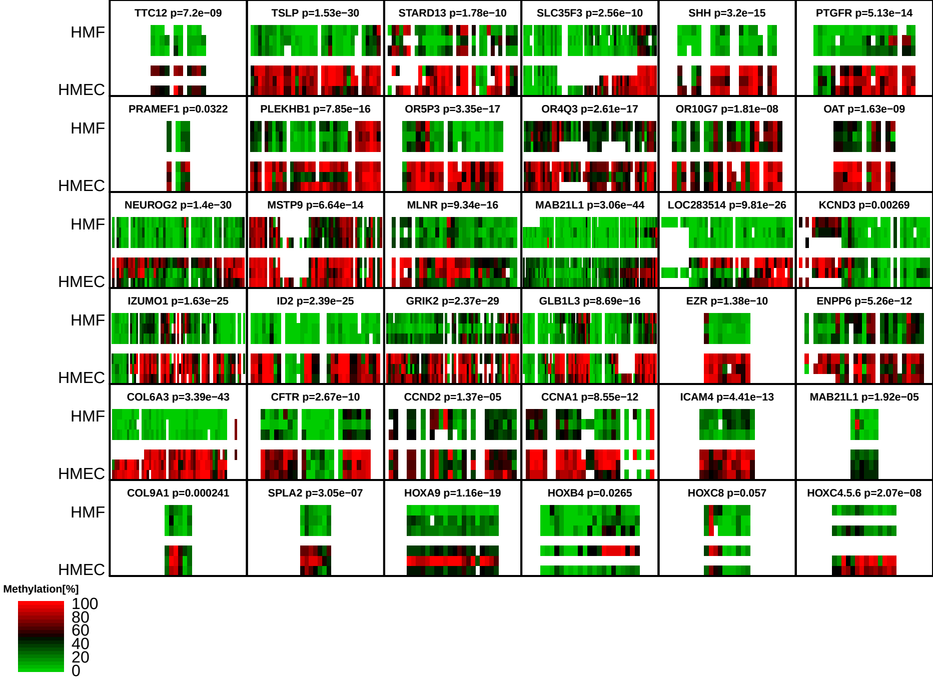

TTC12 p=7.2e−09
TSLP p=1.53e−30
STARD13 p=1.78e−10
SLC35F3 p=2.56e−10
SHH p=3.2e−15
PTGFR p=5.13e−14
PRAMEF1 p=0.0322
PLEKHB1 p=7.85e−16
OR5P3 p=3.35e−17
OR4Q3 p=2.61e−17
OR10G7 p=1.81e−08
OAT p=1.63e−09
NEUROG2 p=1.4e−30
MSTP9 p=6.64e−14
MLNR p=9.34e−16
MAB21L1 p=3.06e−44
LOC283514 p=9.81e−26
KCND3 p=0.00269
IZUMO1 p=1.63e−25
ID2 p=2.39e−25
GRIK2 p=2.37e−29
GLB1L3 p=8.69e−16
EZR p=1.38e−10
ENPP6 p=5.26e−12
COL6A3 p=3.39e−43
CFTR p=2.67e−10
CCND2 p=1.37e−05
CCNA1 p=8.55e−12
ICAM4 p=4.41e−13
MAB21L1 p=1.92e−05
COL9A1 p=0.000241
SPLA2 p=3.05e−07
HOXA9 p=1.16e−19
HOXB4 p=0.0265
HOXC8 p=0.057
HOXC4.5.6 p=2.07e−08
HMF
HMEC
Methylation[%]
100
80
60
40
20
0

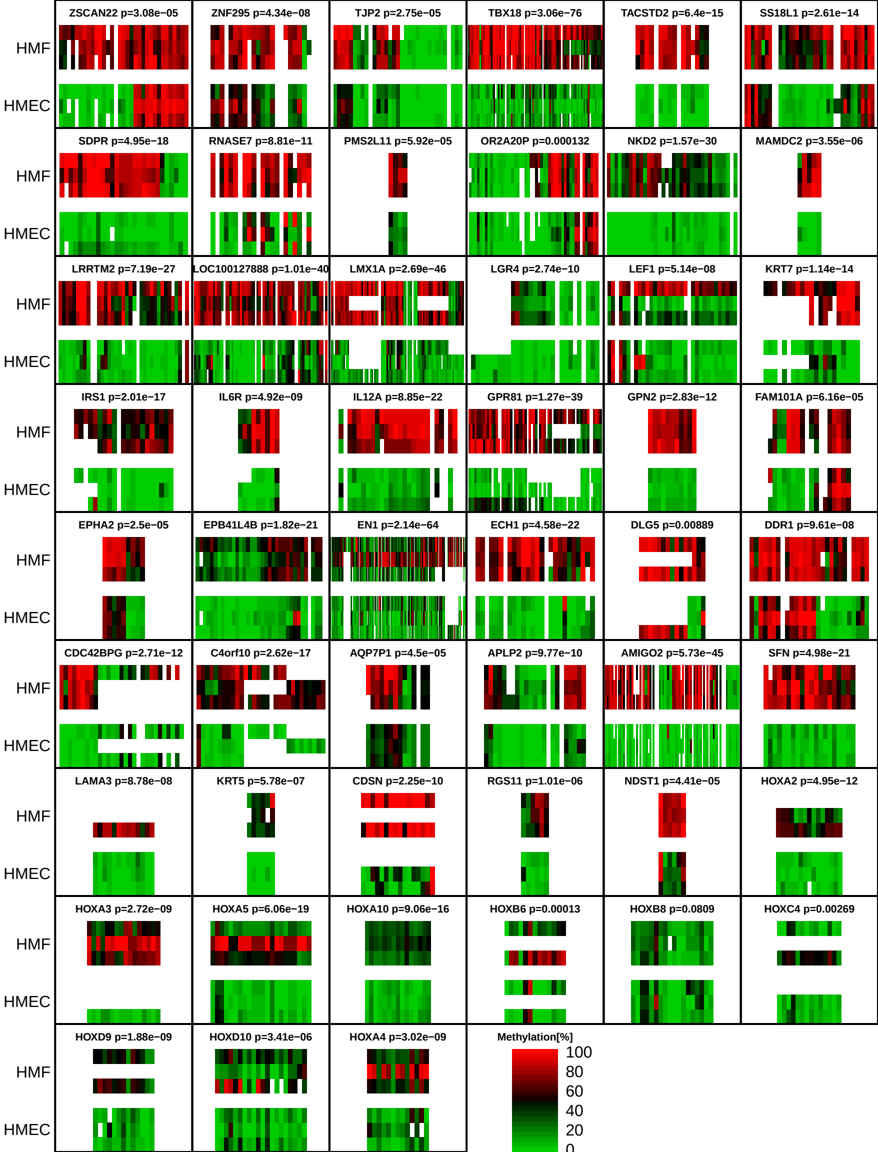
ZSCAN22 p=3.08e−05
ZNF295 p=4.34e−08
TJP2 p=2.75e−05
TBX18 p=3.06e−76
TACSTD2 p=6.4e−15
SS18L1 p=2.61e−14
SDPR p=4.95e−18
RNASE7 p=8.81e−11
PMS2L11 p=5.92e−05
OR2A20P p=0.000132
NKD2 p=1.57e−30
MAMDC2 p=3.55e−06
LRRTM2 p=7.19e−27
LOC100127888 p=1.01e−40
LMX1A p=2.69e−46
LGR4 p=2.74e−10
LEF1 p=5.14e−08
KRT7 p=1.14e−14
IRS1 p=2.01e−17
IL6R p=4.92e−09
IL12A p=8.85e−22
GPR81 p=1.27e−39
GPN2 p=2.83e−12
FAM101A p=6.16e−05
EPHA2 p=2.5e−05
EPB41L4B p=1.82e−21
EN1 p=2.14e−64
ECH1 p=4.58e−22
DLG5 p=0.00889
DDR1 p=9.61e−08
CDC42BPG p=2.71e−12
C4orf10 p=2.62e−17
AQP7P1 p=4.5e−05
APLP2 p=9.77e−10
AMIGO2 p=5.73e−45
SFN p=4.98e−21
LAMA3 p=8.78e−08
KRT5 p=5.78e−07
CDSN p=2.25e−10
RGS11 p=1.01e−06
NDST1 p=4.41e−05
HOXA2 p=4.95e−12
HOXA3 p=2.72e−09
HOXA5 p=6.06e−19
HOXA10 p=9.06e−16
HOXB6 p=0.00013
HOXB8 p=0.0809
HOXC4 p=0.00269
HOXD9 p=1.88e−09
HOXD10 p=3.41e−06
HOXA4 p=3.02e−09
HMF
HMEC
Methylation[%]
100
80
60
40
20
0

AMIGO2

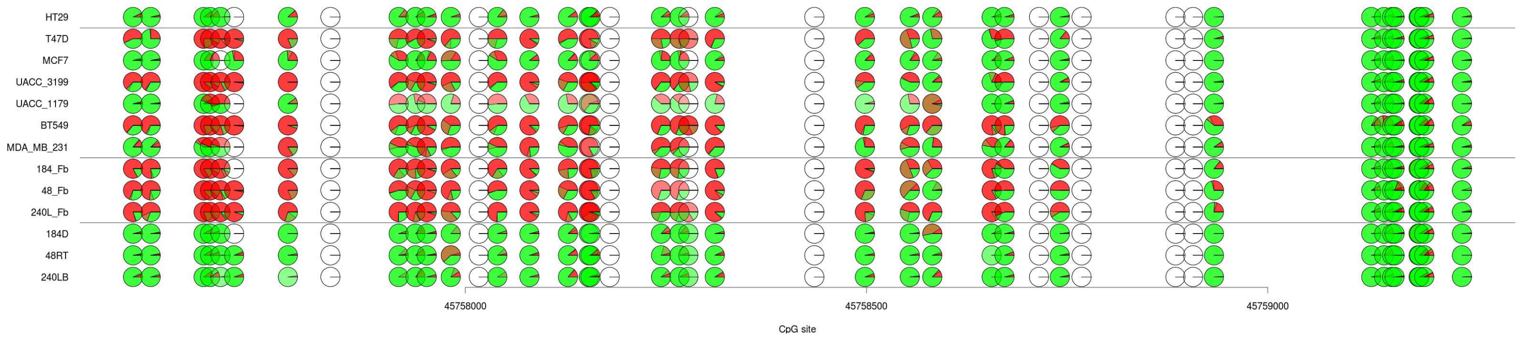

HT29
T47D
MCF7
UACC_3199
UACC_1179
BT549
MDA_MB_231
184_Fb
48_Fb
240L_Fb
184D
48RT
240LB
45758000
45758500
45759000
CpG site

APLP2

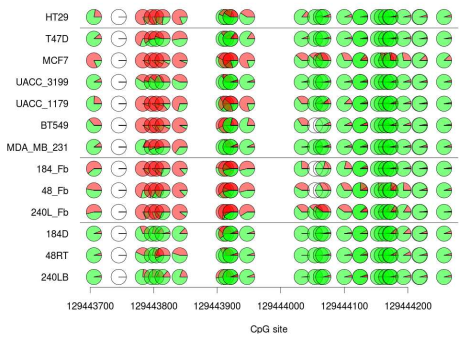
HT29
T47D
MCF7
UACC_3199
UACC_1179
BT549
MDA_MB_231
184_Fb
48_Fb
240L_Fb
184D
48RT
240LB
129443700
129443800
129443900
129444000
129444100
129444200
CpG site

AQP7P1

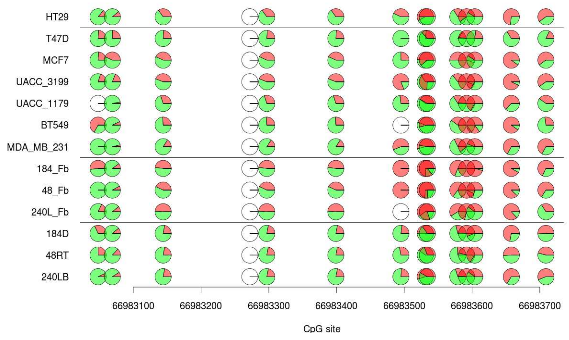
HT29
T47D
MCF7
UACC_3199
UACC_1179
BT549
MDA_MB_231
184_Fb
48_Fb
240L_Fb
184D
48RT
240LB
66983100
66983200
66983300
66983400
66983500
66983600
66983700
CpG site

C4orf10

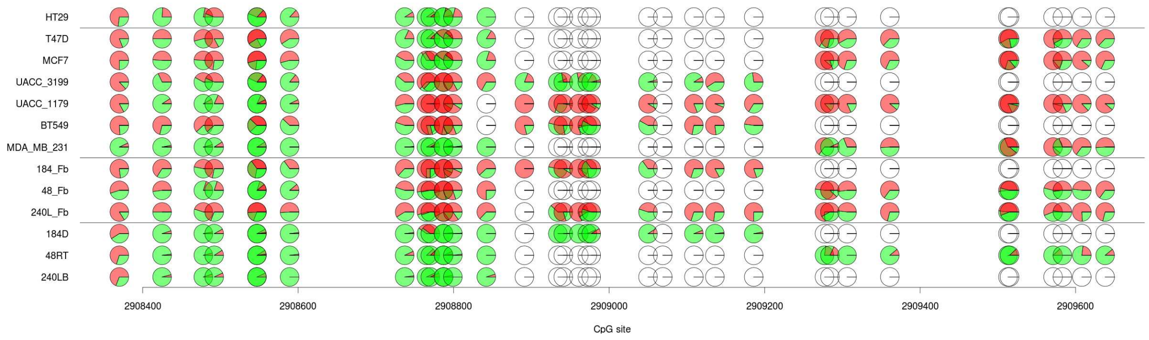
HT29
T47D
MCF7
UACC_3199
UACC_1179
BT549
MDA_MB_231
184_Fb
48_Fb
240L_Fb
184D
48RT
240LB
2908400
2908600
2908800
2909000
2909200
2909400
2909600
CpG site

CCNA1

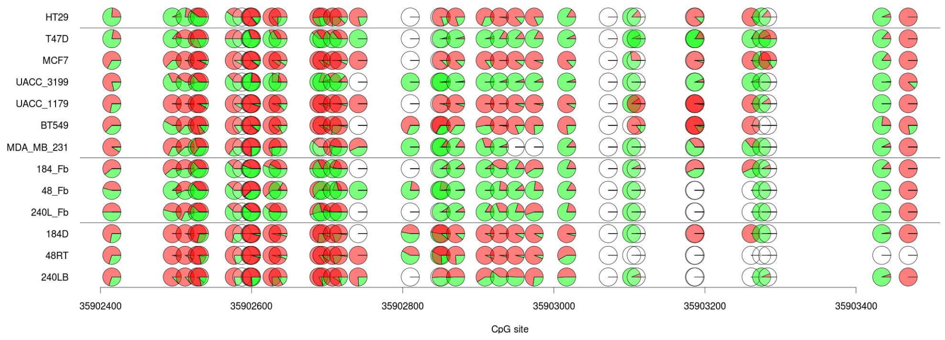
HT29
T47D
MCF7
UACC_3199
UACC_1179
BT549
MDA_MB_231
184_Fb
48_Fb
240L_Fb
184D
48RT
240LB
35902400
35902600
35902800
35903000
35903200
35903400
CpG site

CCND2

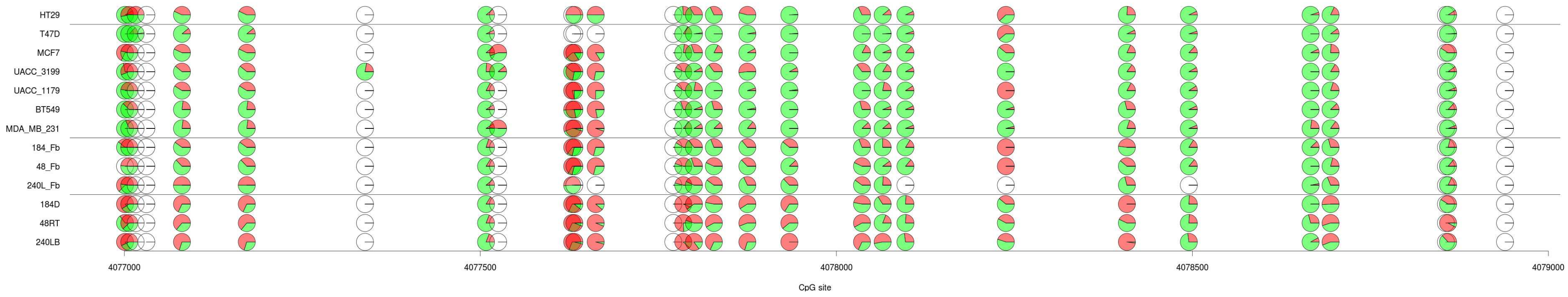
HT29
T47D
MCF7
UACC_3199
UACC_1179
BT549
MDA_MB_231
184_Fb
48_Fb
240L_Fb
184D
48RT
240LB
4077000
4077500
4078000
4078500
4079000
CpG site

CDC42BPG

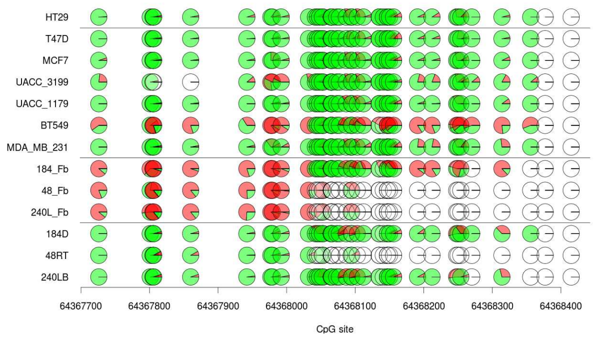
HT29
T47D
MCF7
UACC_3199
UACC_1179
BT549
MDA_MB_231
184_Fb
48_Fb
240L_Fb
184D
48RT
240LB
64367700
64367800
64367900
64368000
64368100
64368200
64368300
64368400
CpG site

CFTR

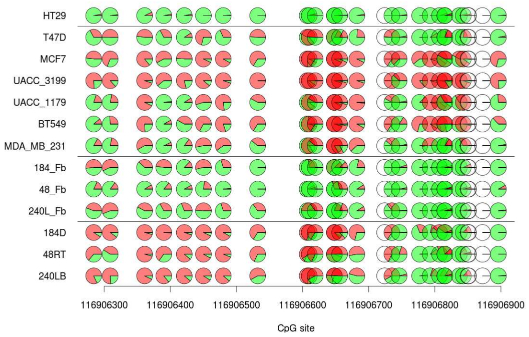

HT29
T47D
MCF7
UACC_3199
UACC_1179
BT549
MDA_MB_231
184_Fb
48_Fb
240L_Fb
184D
48RT
240LB
116906300
116906400
116906500
116906600
116906700
116906800
116906900
CpG site

COL6A3

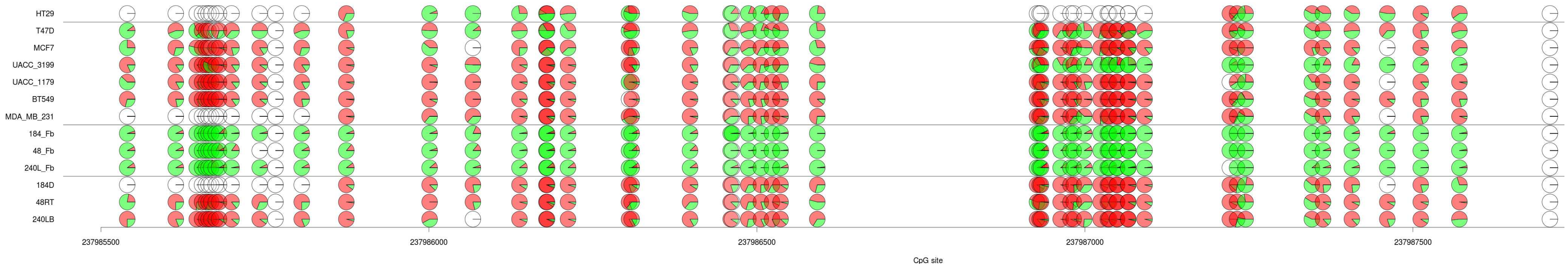

HT29
T47D
MCF7
UACC_3199
UACC_1179
BT549
MDA_MB_231
184_Fb
48_Fb
240L_Fb
184D
48RT
240LB
237985500
237986000
237986500
237987000
237987500
CpG site

DDR1

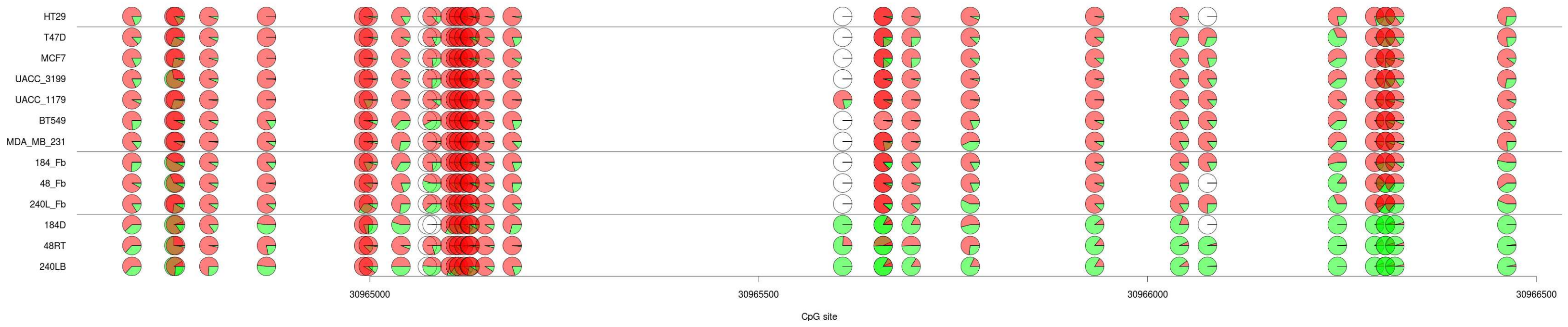
HT29
T47D
MCF7
UACC_3199
UACC_1179
BT549
MDA_MB_231
184_Fb
48_Fb
240L_Fb
184D
48RT
240LB
30965000
30965500
30966000
30966500
CpG site

DLG5

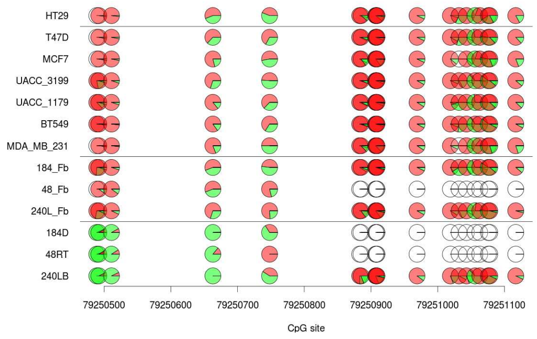
HT29
T47D
MCF7
UACC_3199
UACC_1179
BT549
MDA_MB_231
184_Fb
48_Fb
240L_Fb
184D
48RT
240LB
79250500
79250600
79250700
79250800
79250900
79251000
79251100
CpG site

ECH1

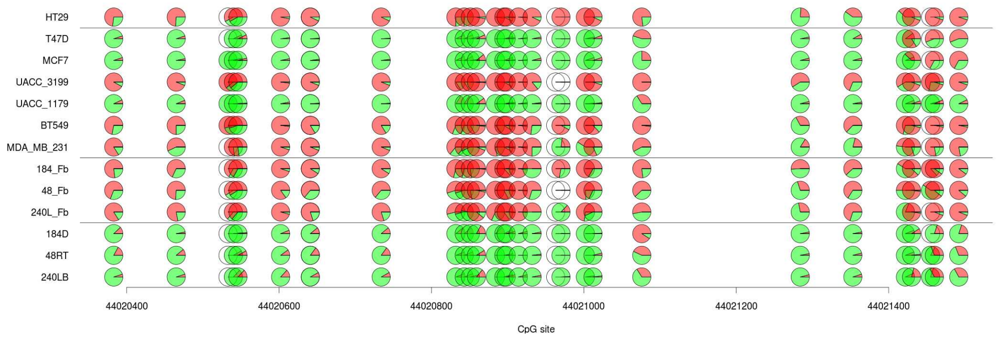
HT29
T47D
MCF7
UACC_3199
UACC_1179
BT549
MDA_MB_231
184_Fb
48_Fb
240L_Fb
184D
48RT
240LB
44020400
44020600
44020800
44021000
44021200
44021400
CpG site

EN1

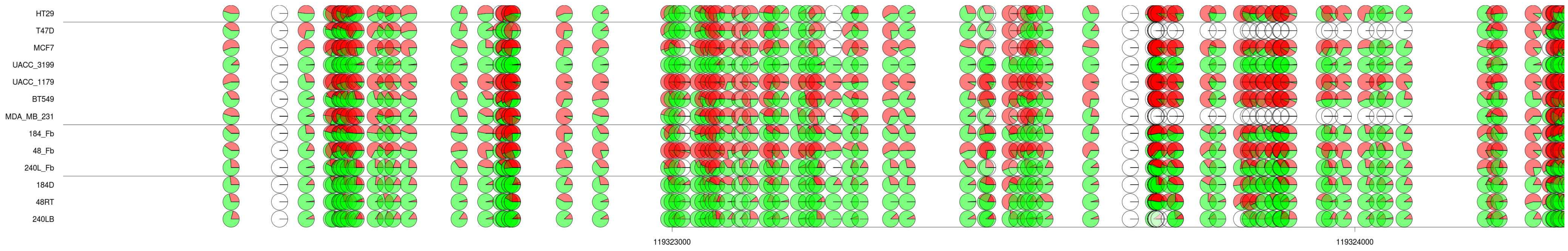
HT29
T47D
MCF7
UACC_3199
UACC_1179
BT549
MDA_MB_231
184_Fb
48_Fb
240L_Fb
184D
48RT
240LB
119323000
119324000

ENPP6

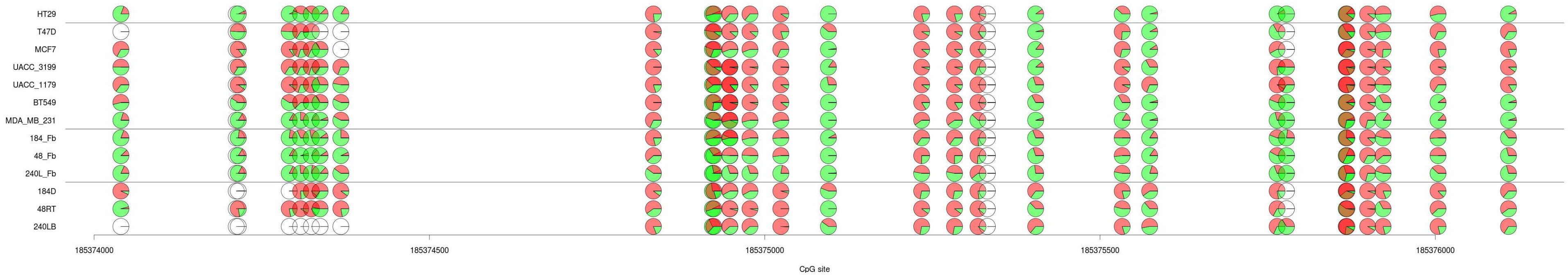

HT29
T47D
MCF7
UACC_3199
UACC_1179
BT549
MDA_MB_231
184_Fb
48_Fb
240L_Fb
184D
48RT
240LB
185374000
185374500
185375000
185375500
185376000
CpG site

EPB41L4B

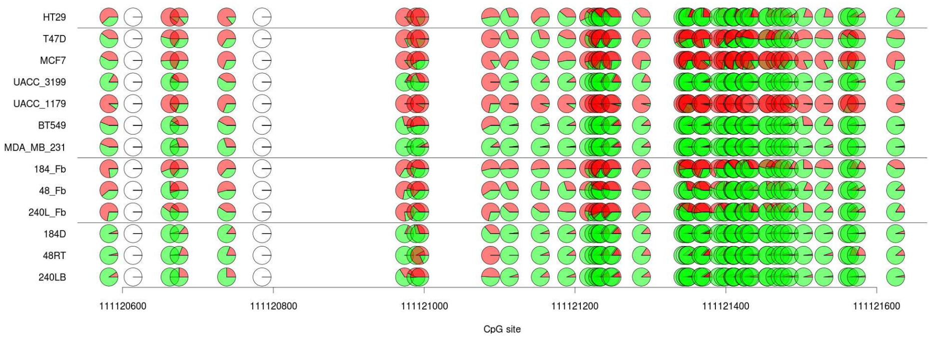
HT29
T47D
MCF7
UACC_3199
UACC_1179
BT549
MDA_MB_231
184_Fb
48_Fb
240L_Fb
184D
48RT
240LB
111120600
111120800
111121000
111121200
111121400
111121600
CpG site

EPHA2

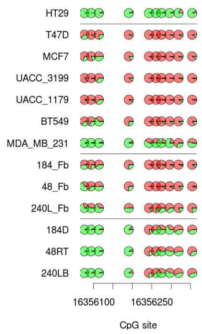
HT29
T47D
MCF7
UACC_3199
UACC_1179
BT549
MDA_MB_231
184_Fb
48_Fb
240L_Fb
184D
48RT
240LB
16356100
16356250
CpG site

EZR

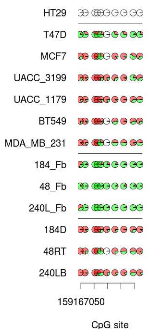
HT29
T47D
MCF7
UACC_3199
UACC_1179
BT549
MDA_MB_231
184_Fb
48_Fb
240L_Fb
184D
48RT
240LB
159167050
CpG site

FAM101A

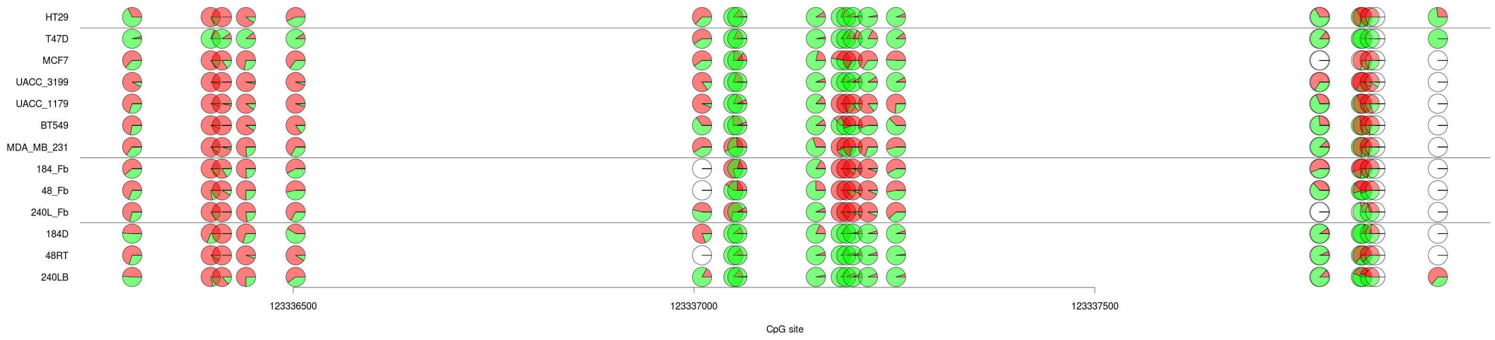

HT29
T47D
MCF7
UACC_3199
UACC_1179
BT549
MDA_MB_231
184_Fb
48_Fb
240L_Fb
184D
48RT
240LB
123336500
123337000
123337500
CpG site

GLB1L3

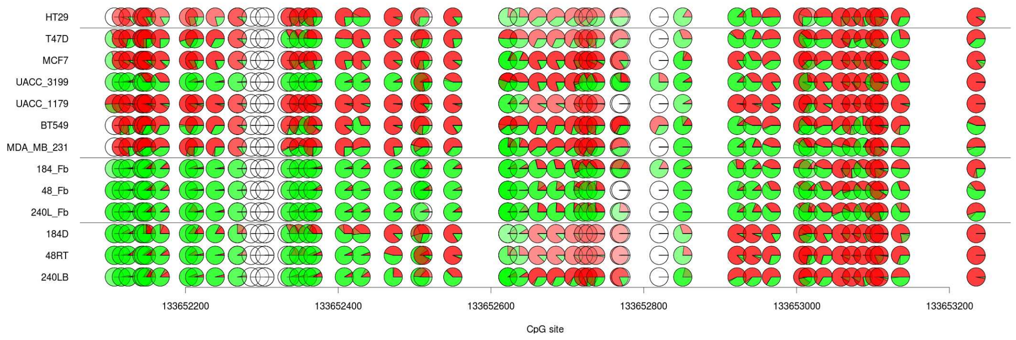

HT29
T47D
MCF7
UACC_3199
UACC_1179
BT549
MDA_MB_231
184_Fb
48_Fb
240L_Fb
184D
48RT
240LB
133652200
133652400
133652600
133652800
133653000
133653200
CpG site

GPN2

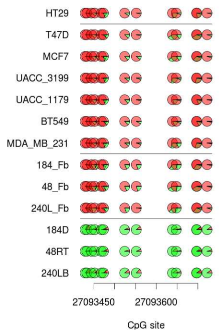
HT29
T47D
MCF7
UACC_3199
UACC_1179
BT549
MDA_MB_231
184_Fb
48_Fb
240L_Fb
184D
48RT
240LB
27093450
27093600
CpG site

GPR81

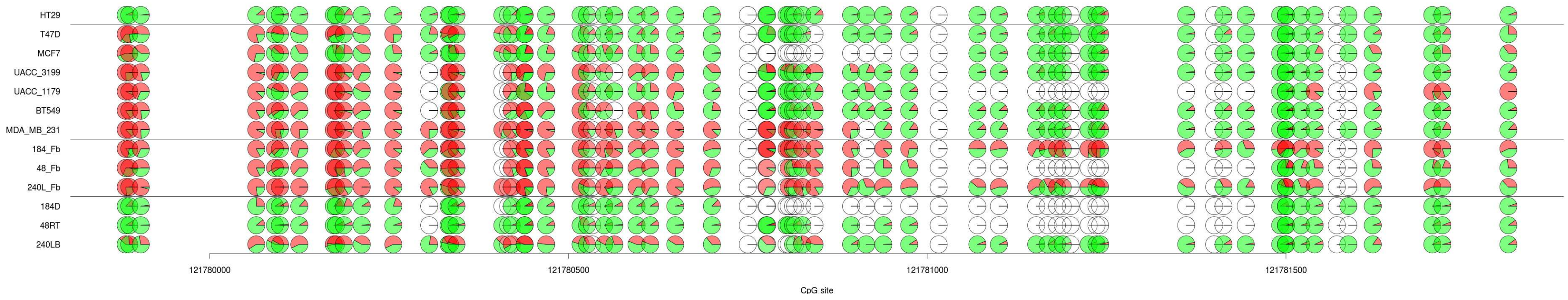

HT29
T47D
MCF7
UACC_3199
UACC_1179
BT549
MDA_MB_231
184_Fb
48_Fb
240L_Fb
184D
48RT
240LB
121780000
121780500
121781000
121781500
CpG site

GRIK2

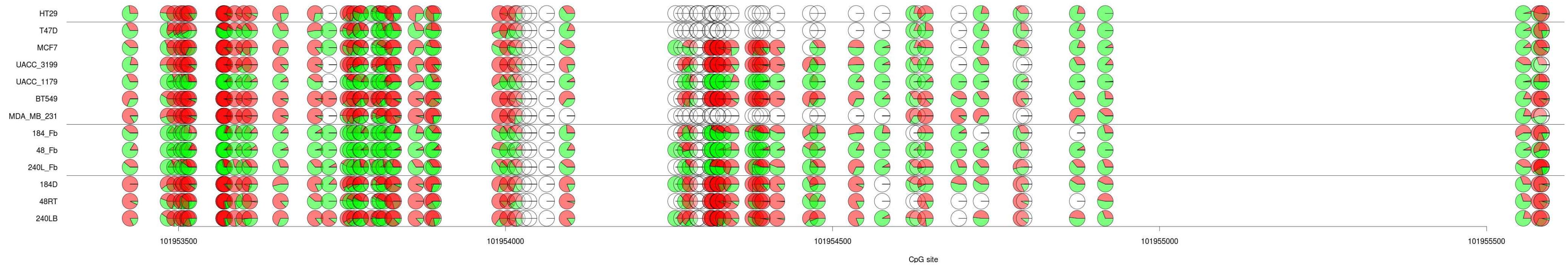

HT29
T47D
MCF7
UACC_3199
UACC_1179
BT549
MDA_MB_231
184_Fb
48_Fb
240L_Fb
184D
48RT
240LB
101953500
101954000
101954500
101955000
101955500
CpG site

ID2

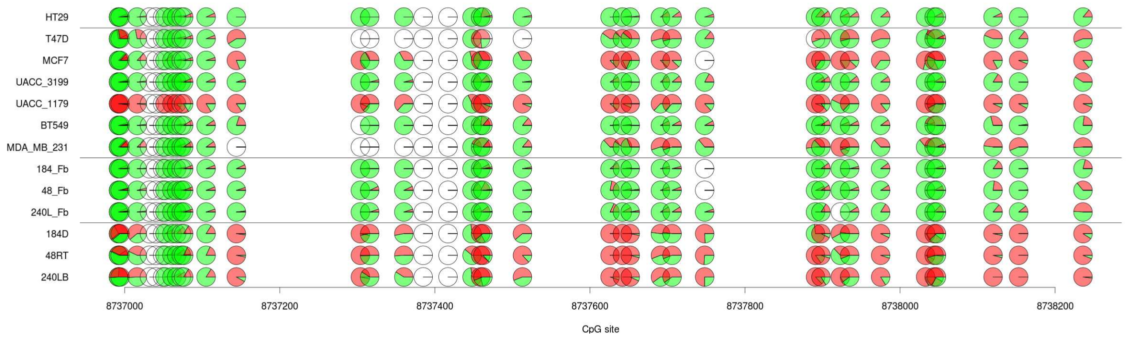

HT29
T47D
MCF7
UACC_3199
UACC_1179
BT549
MDA_MB_231
184_Fb
48_Fb
240L_Fb
184D
48RT
240LB
8737000
8737200
8737400
8737600
8737800
8738000
8738200
CpG site

IL12A

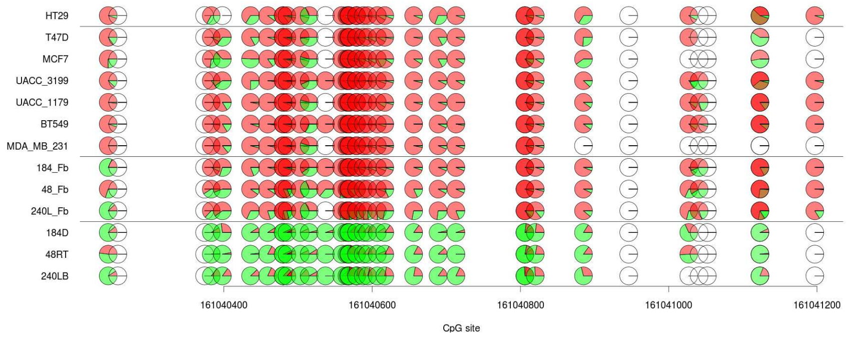
HT29
T47D
MCF7
UACC_3199
UACC_1179
BT549
MDA_MB_231
184_Fb
48_Fb
240L_Fb
184D
48RT
240LB
161040400
161040600
161040800
161041000
161041200
CpG site

IL6R

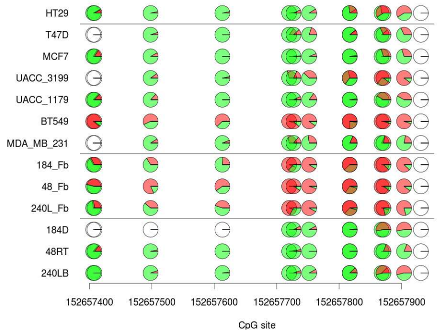
HT29
T47D
MCF7
UACC_3199
UACC_1179
BT549
MDA_MB_231
184_Fb
48_Fb
240L_Fb
184D
48RT
240LB
152657400
152657500
152657600
152657700
152657800
152657900
CpG site

IRS1

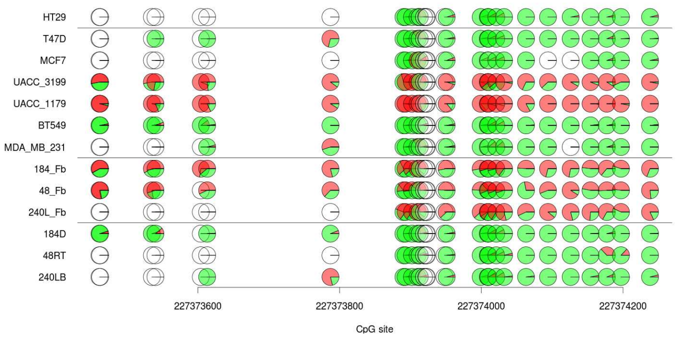

HT29
T47D
MCF7
UACC_3199
UACC_1179
BT549
MDA_MB_231
184_Fb
48_Fb
240L_Fb
184D
48RT
240LB
227373600
227373800
227374000
227374200
CpG site

IZUMO1

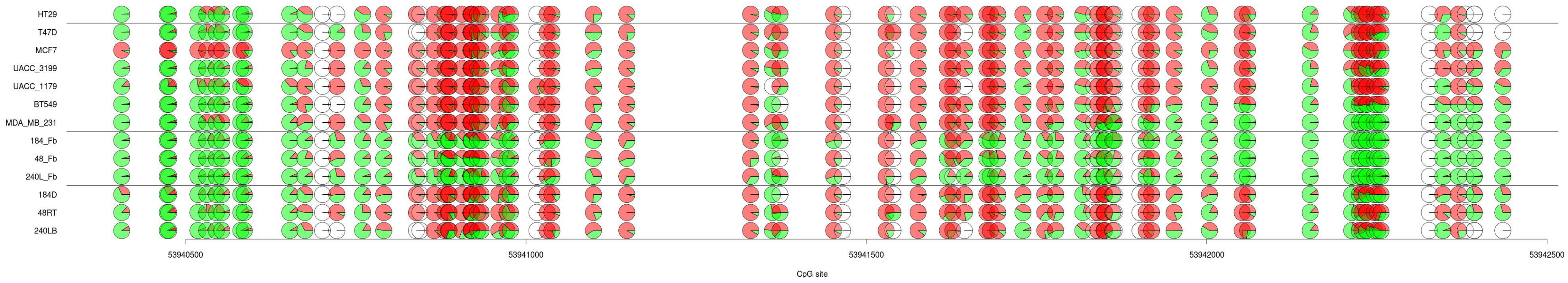
HT29
T47D
MCF7
UACC_3199
UACC_1179
BT549
MDA_MB_231
184_Fb
48_Fb
240L_Fb
184D
48RT
240LB
53940500
53941000
53941500
53942000
53942500
CpG site

KCND3

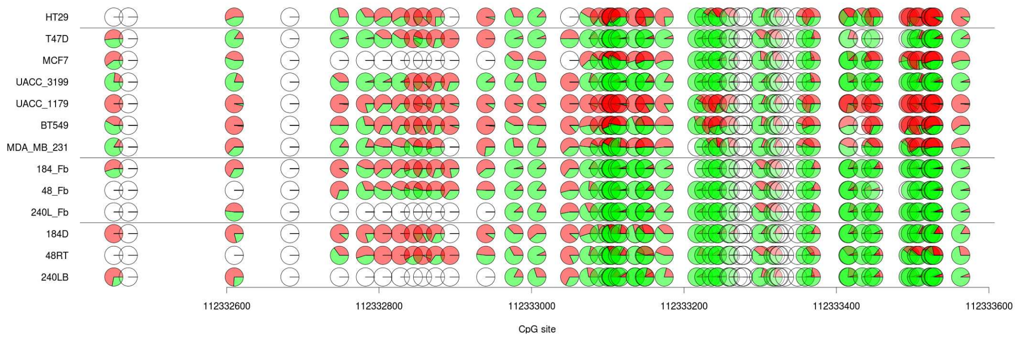

HT29
T47D
MCF7
UACC_3199
UACC_1179
BT549
MDA_MB_231
184_Fb
48_Fb
240L_Fb
184D
48RT
240LB
112332600
112332800
112333000
112333200
112333400
112333600
CpG site

KRT7_1

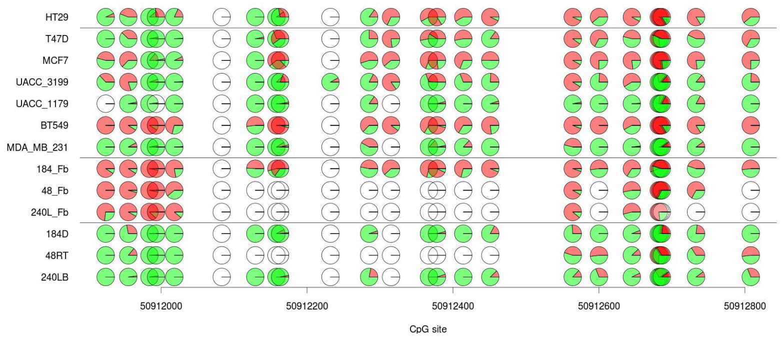

HT29
T47D
MCF7
UACC_3199
UACC_1179
BT549
MDA_MB_231
184_Fb
48_Fb
240L_Fb
184D
48RT
240LB
50912000
50912200
50912400
50912600
50912800
CpG site

LEF1

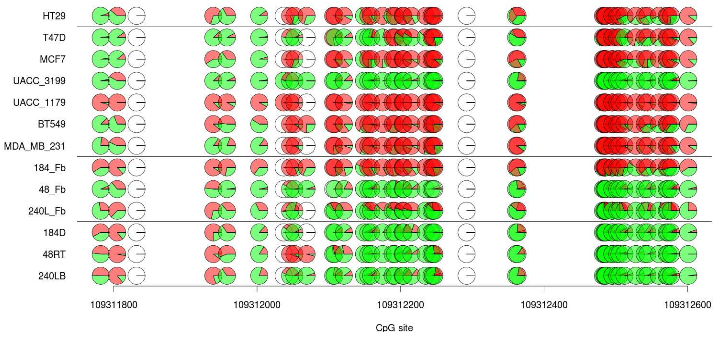

HT29
T47D
MCF7
UACC_3199
UACC_1179
BT549
MDA_MB_231
184_Fb
48_Fb
240L_Fb
184D
48RT
240LB
109311800
109312000
109312200
109312400
109312600
CpG site

LGR4

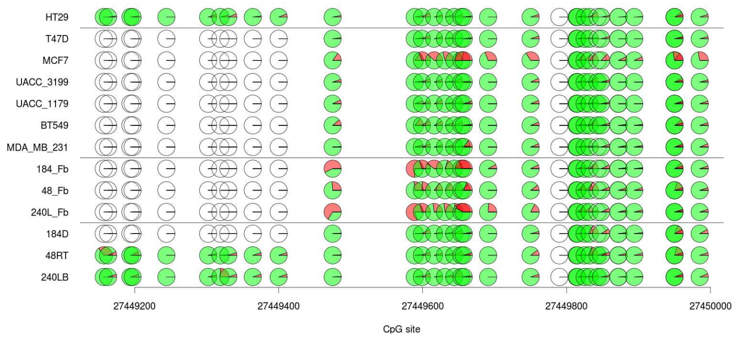
HT29
T47D
MCF7
UACC_3199
UACC_1179
BT549
MDA_MB_231
184_Fb
48_Fb
240L_Fb
184D
48RT
240LB
27449200
27449400
27449600
27449800
27450000
CpG site

LMX1A

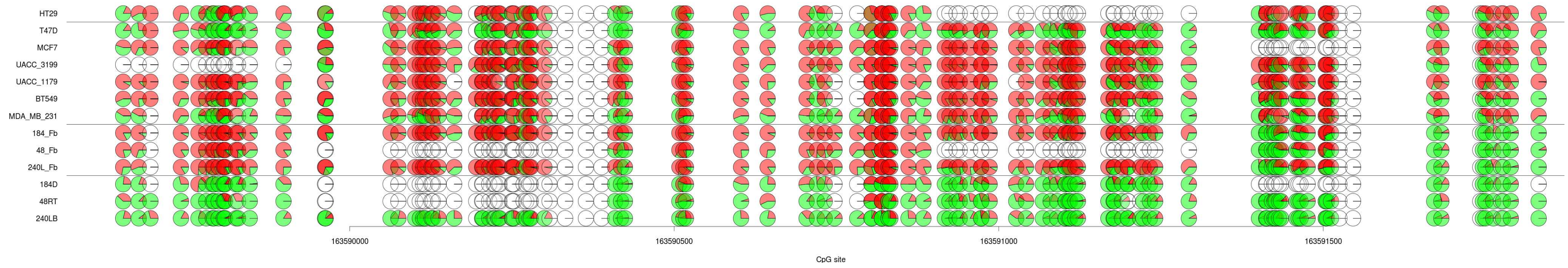
HT29
T47D
MCF7
UACC_3199
UACC_1179
BT549
MDA_MB_231
184_Fb
48_Fb
240L_Fb
184D
48RT
240LB
163590000
163590500
163591000
163591500
CpG site

LOC100127888

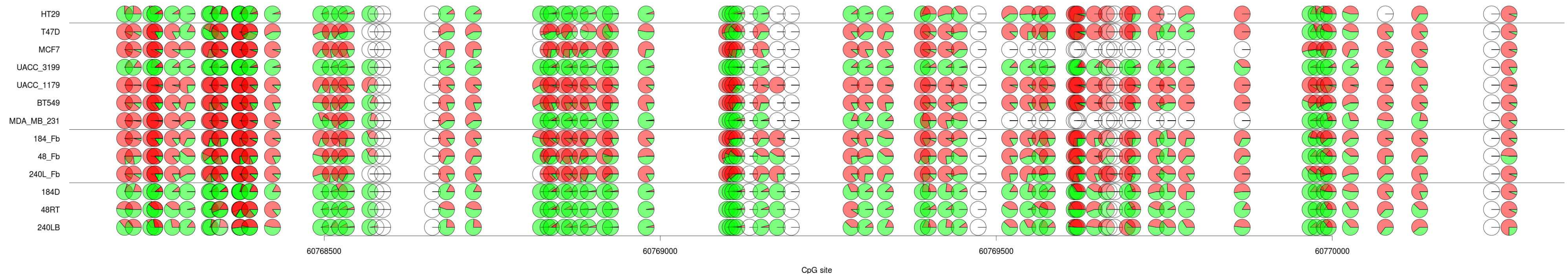

LOC283514

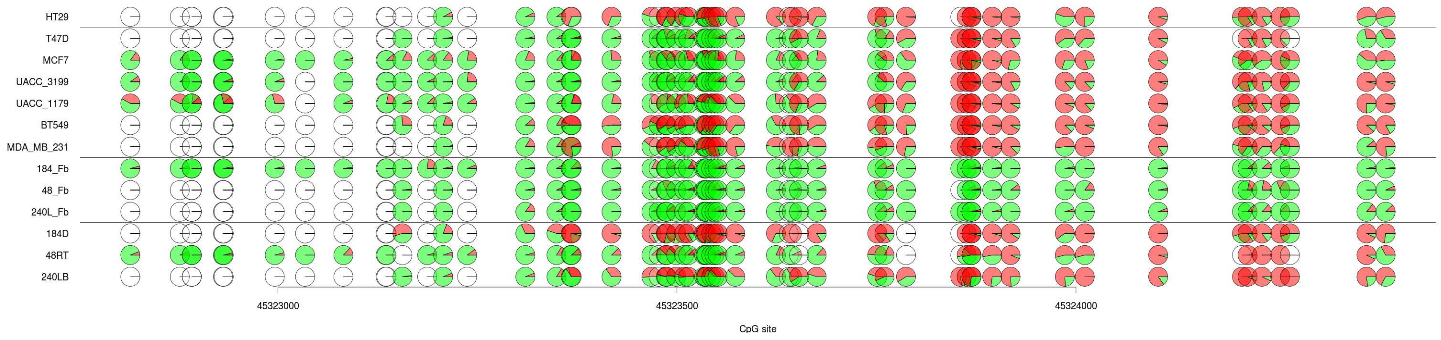

HT29
T47D
MCF7
UACC_3199
UACC_1179
BT549
MDA_MB_231
184_Fb
48_Fb
240L_Fb
184D
48RT
240LB
45323000
45323500
45324000
CpG site

LRRTM2

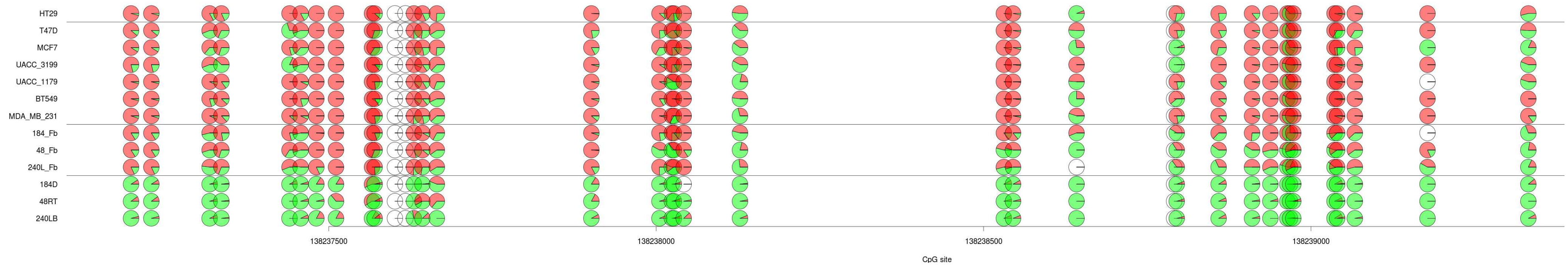

HT29
T47D
MCF7
UACC_3199
UACC_1179
BT549
MDA_MB_231
184_Fb
48_Fb
240L_Fb
184D
48RT
240LB
138237500
138238000
138238500
138239000
CpG site

MAB21L1

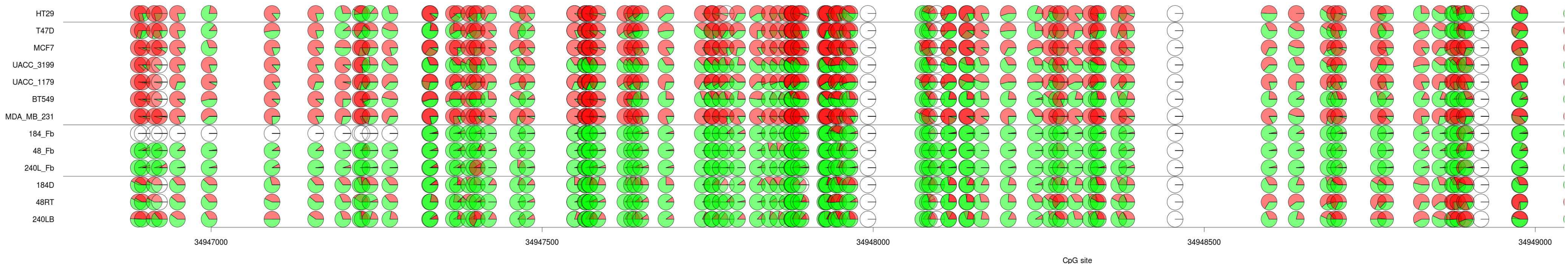
HT29
T47D
MCF7
UACC_3199
UACC_1179
BT549
MDA_MB_231
184_Fb
48_Fb
240L_Fb
184D
48RT
240LB
34947000
34947500
34948000
34948500
34949000
CpG site

MAMDC2

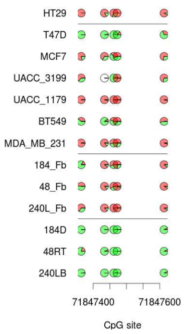

HT29
T47D
MCF7
UACC_3199
UACC_1179
BT549
MDA_MB_231
184_Fb
48_Fb
240L_Fb
184D
48RT
240LB
71847400
71847600
CpG site

MLNR

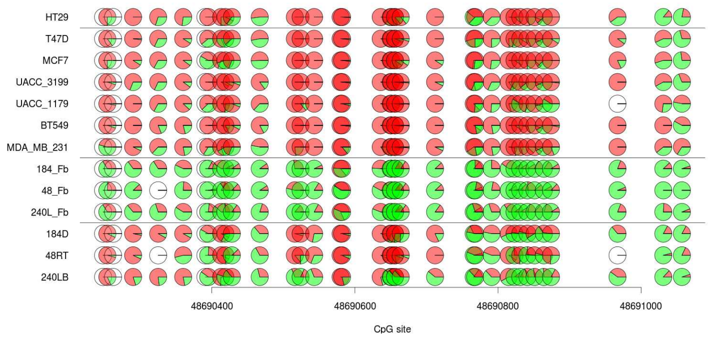

HT29
T47D
MCF7
UACC_3199
UACC_1179
BT549
MDA_MB_231
184_Fb
48_Fb
240L_Fb
184D
48RT
240LB
48690400
48690600
48690800
48691000
CpG site

MSTP9

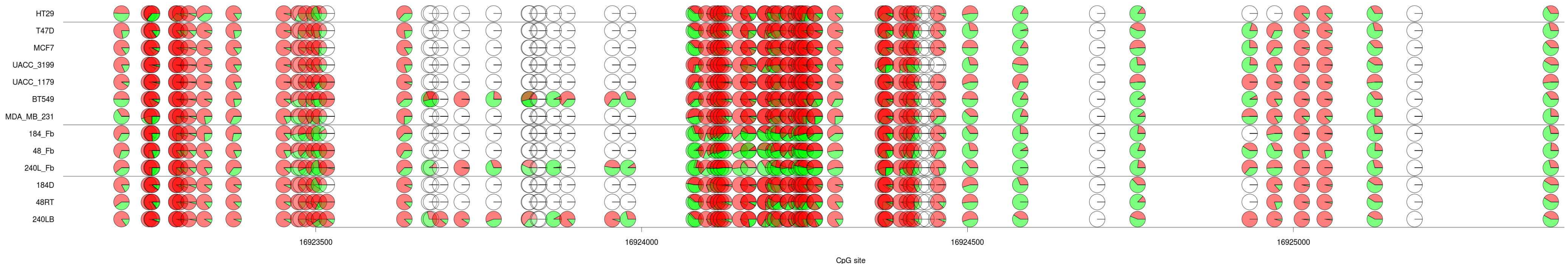
HT29
T47D
MCF7
UACC_3199
UACC_1179
BT549
MDA_MB_231
184_Fb
48_Fb
240L_Fb
184D
48RT
240LB
16923500
16924000
16924500
16925000
CpG site

NEUROG2

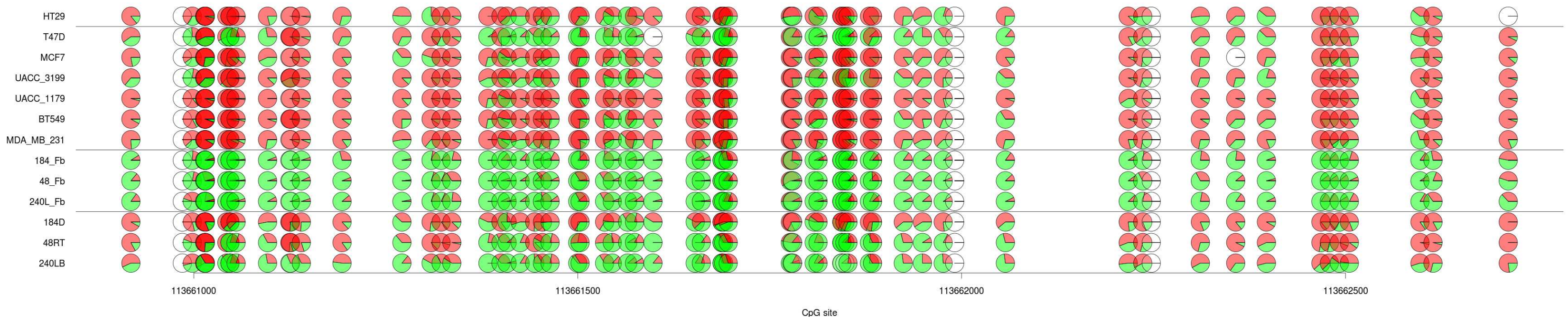
HT29
T47D
MCF7
UACC_3199
UACC_1179
BT549
MDA_MB_231
184_Fb
48_Fb
240L_Fb
184D
48RT
240LB
113661000
113661500
113662000
113662500
CpG site

NKD2

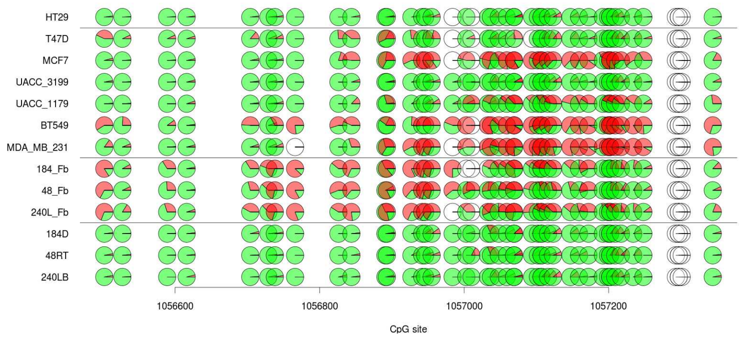
HT29
T47D
MCF7
UACC_3199
UACC_1179
BT549
MDA_MB_231
184_Fb
48_Fb
240L_Fb
184D
48RT
240LB
1056600
1056800
1057000
1057200
CpG site

OAT

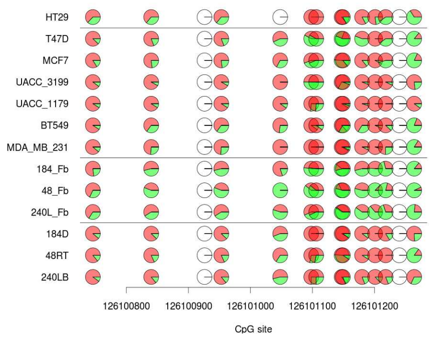
HT29
T47D
MCF7
UACC_3199
UACC_1179
BT549
MDA_MB_231
184_Fb
48_Fb
240L_Fb
184D
48RT
240LB
126100800
126100900
126101000
126101100
126101200
CpG site

OR10G7

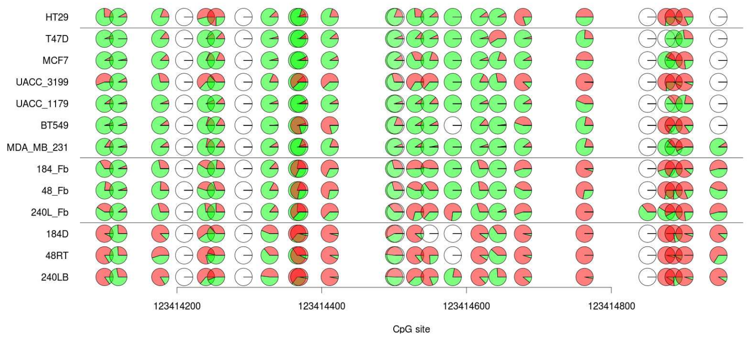

HT29
T47D
MCF7
UACC_3199
UACC_1179
BT549
MDA_MB_231
184_Fb
48_Fb
240L_Fb
184D
48RT
240LB
123414200
123414400
123414600
123414800
CpG site

OR2A20P

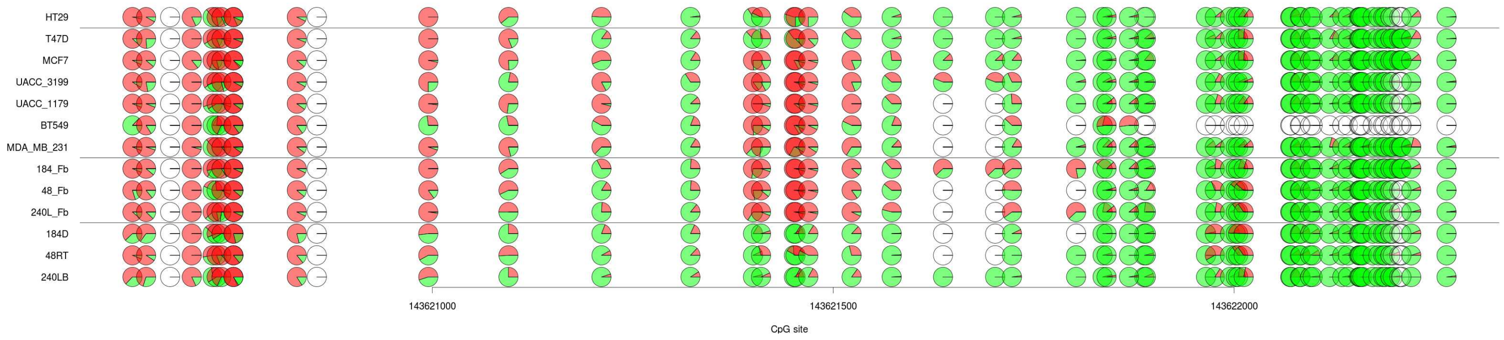
HT29
T47D
MCF7
UACC_3199
UACC_1179
BT549
MDA_MB_231
184_Fb
48_Fb
240L_Fb
184D
48RT
240LB
143621000
143621500
143622000
CpG site

OR4Q3

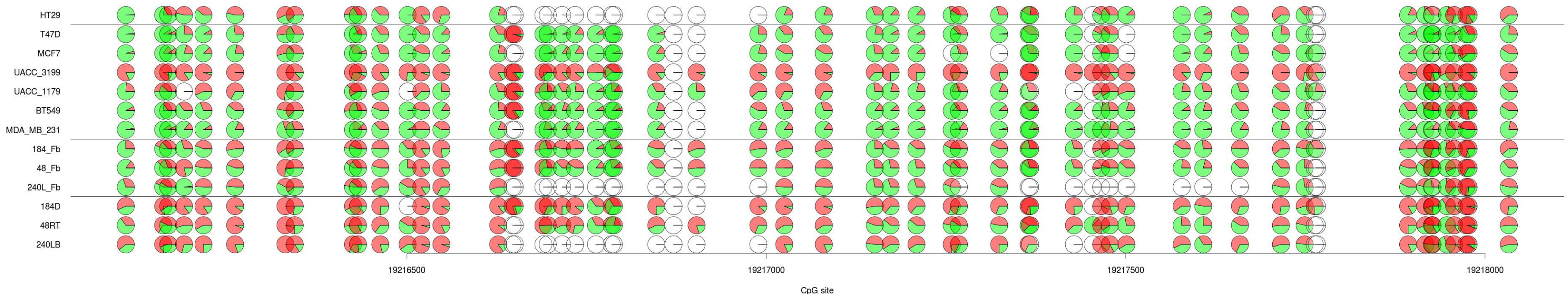

HT29
T47D
MCF7
UACC_3199
UACC_1179
BT549
MDA_MB_231
184_Fb
48_Fb
240L_Fb
184D
48RT
240LB
19216500
19217000
19217500
19218000
CpG site

OR5P3

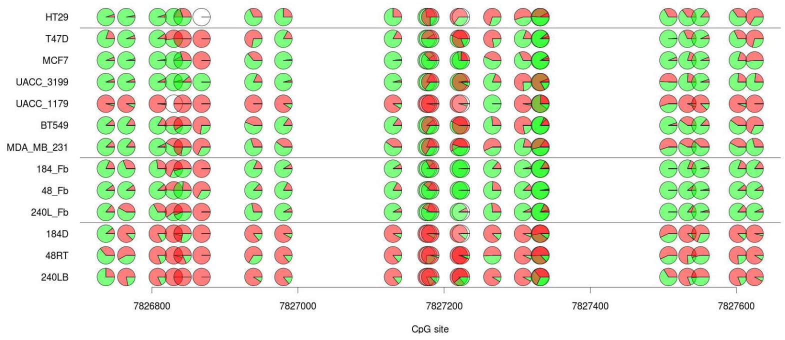

HT29
T47D
MCF7
UACC_3199
UACC_1179
BT549
MDA_MB_231
184_Fb
48_Fb
240L_Fb
184D
48RT
240LB
7826800
7827000
7827200
7827400
7827600
CpG site

PLEKHB1

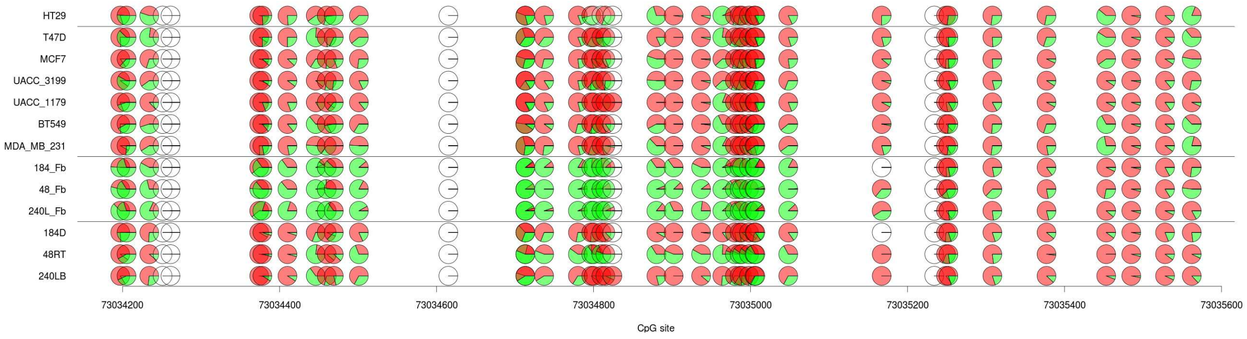
HT29
T47D
MCF7
UACC_3199
UACC_1179
BT549
MDA_MB_231
184_Fb
48_Fb
240L_Fb
184D
48RT
240LB
73034200
73034400
73034600
73034800
73035000
73035200
73035400
73035600
CpG site

PMS2L11

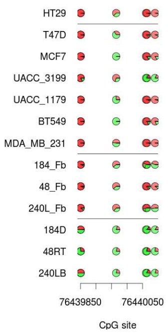
HT29
T47D
MCF7
UACC_3199
UACC_1179
BT549
MDA_MB_231
184_Fb
48_Fb
240L_Fb
184D
48RT
240LB
76439850
76440050
CpG site

PRAMEF1

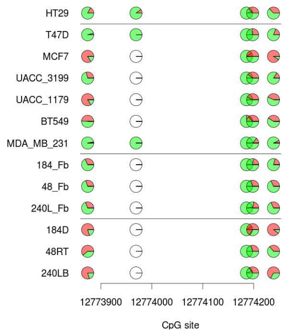

HT29
T47D
MCF7
UACC_3199
UACC_1179
BT549
MDA_MB_231
184_Fb
48_Fb
240L_Fb
184D
48RT
240LB
12773900
12774000
12774100
12774200
CpG site

PTGFR

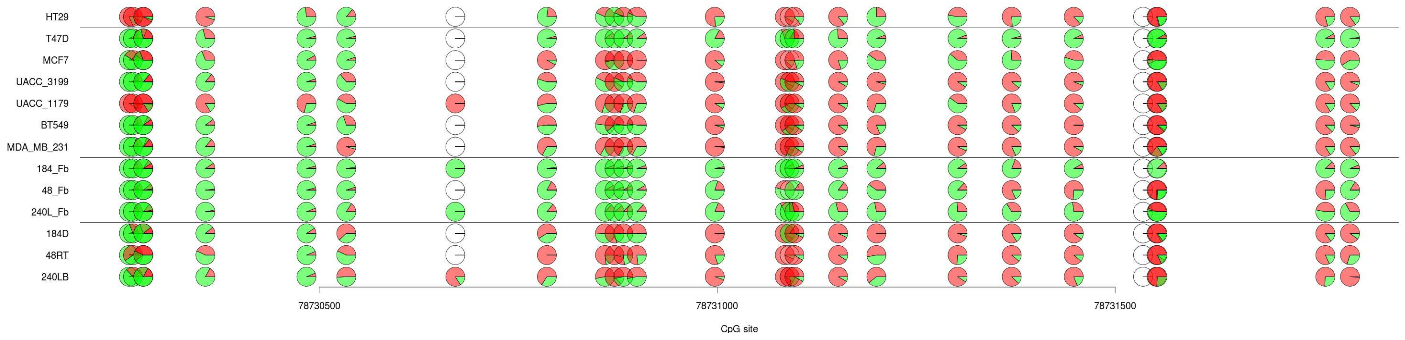
HT29
T47D
MCF7
UACC_3199
UACC_1179
BT549
MDA_MB_231
184_Fb
48_Fb
240L_Fb
184D
48RT
240LB
78730500
78731000
78731500
CpG site

RNASE7

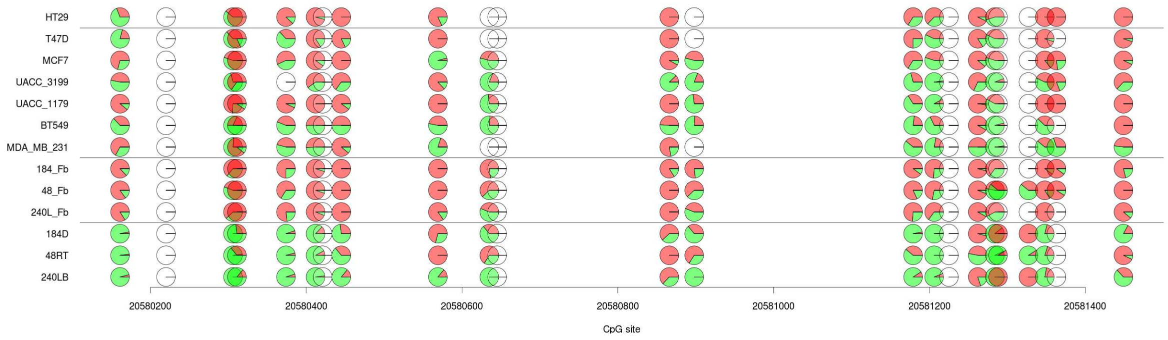

HT29
T47D
MCF7
UACC_3199
UACC_1179
BT549
MDA_MB_231
184_Fb
48_Fb
240L_Fb
184D
48RT
240LB
20580200
20580400
20580600
20580800
20581000
20581200
20581400
CpG site

SDPR

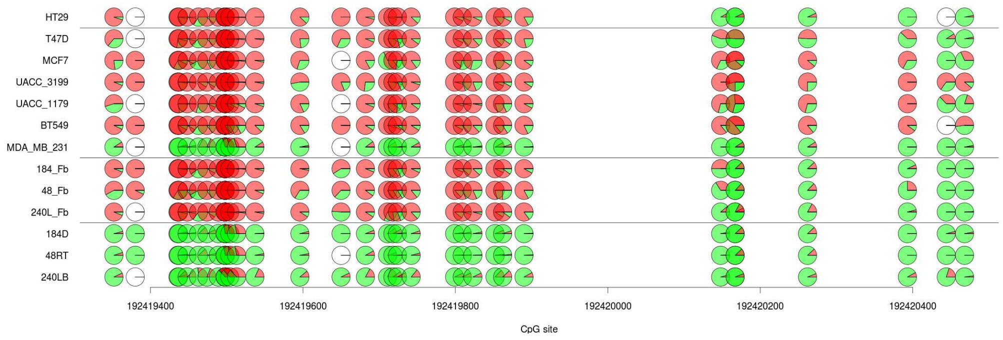
HT29
T47D
MCF7
UACC_3199
UACC_1179
BT549
MDA_MB_231
184_Fb
48_Fb
240L_Fb
184D
48RT
240LB
192419400
192419600
192419800
192420000
192420200
192420400
CpG site

SHH

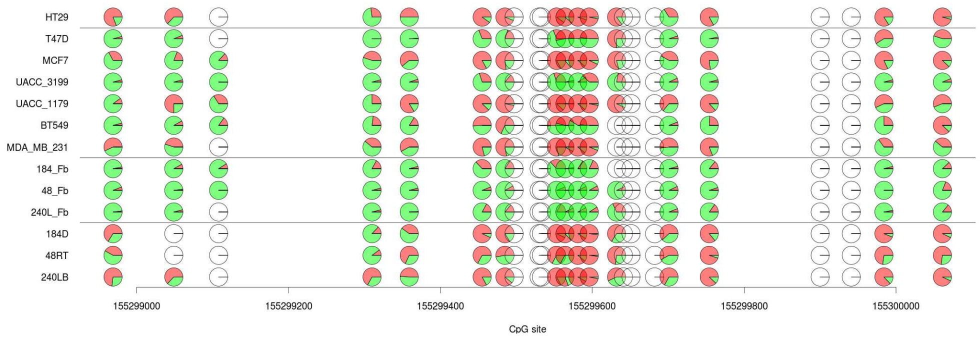
HT29
T47D
MCF7
UACC_3199
UACC_1179
BT549
MDA_MB_231
184_Fb
48_Fb
240L_Fb
184D
48RT
240LB
155299000
155299200
155299400
155299600
155299800
155300000
CpG site

SLC35F3

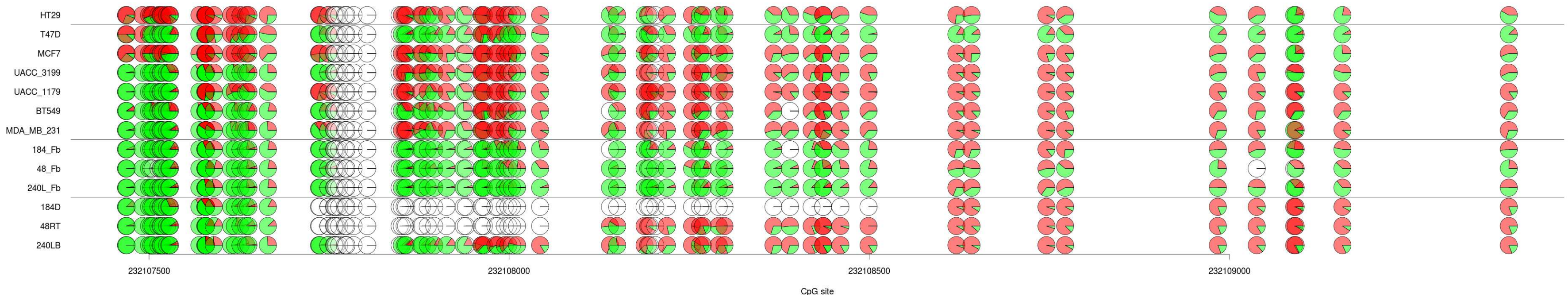
HT29
T47D
MCF7
UACC_3199
UACC_1179
BT549
MDA_MB_231
184_Fb
48_Fb
240L_Fb
184D
48RT
240LB
232107500
232108000
232108500
232109000
CpG site

SS18L1

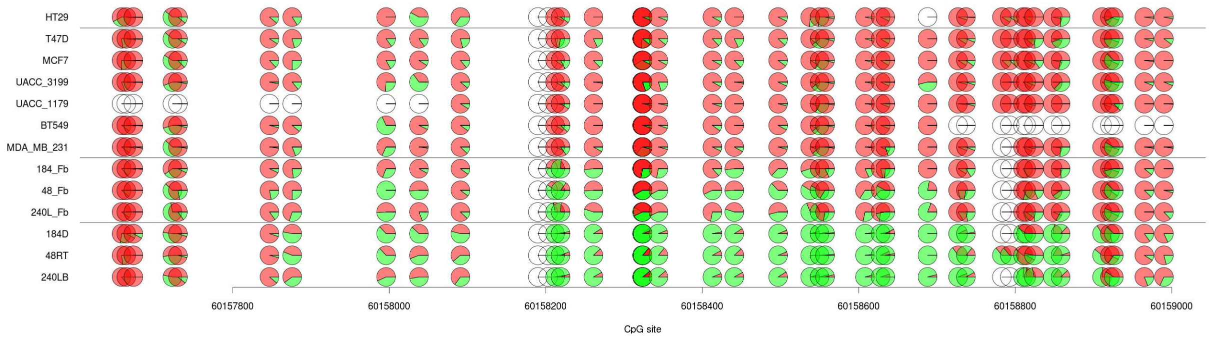

HT29
T47D
MCF7
UACC_3199
UACC_1179
BT549
MDA_MB_231
184_Fb
48_Fb
240L_Fb
184D
48RT
240LB
60157800
60158000
60158200
60158400
60158600
60158800
60159000
CpG site

STARD13

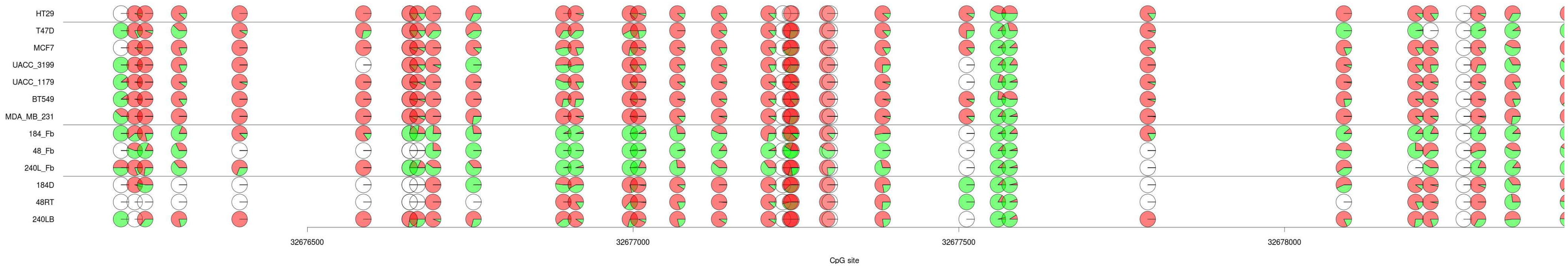
HT29
T47D
MCF7
UACC_3199
UACC_1179
BT549
MDA_MB_231
184_Fb
48_Fb
240L_Fb
184D
48RT
240LB
32676500
32677000
32677500
32678000
CpG site

TACSTD2

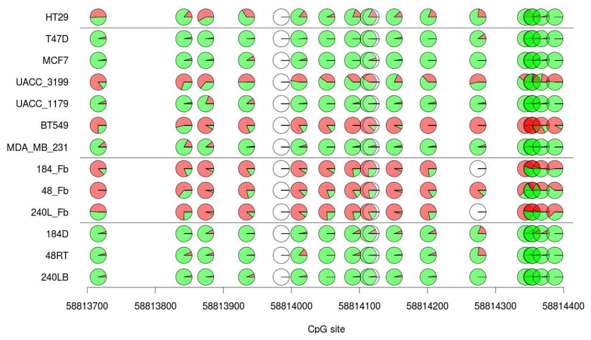

HT29
T47D
MCF7
UACC_3199
UACC_1179
BT549
MDA_MB_231
184_Fb
48_Fb
240L_Fb
184D
48RT
240LB
58813700
58813800
58813900
58814000
58814100
58814200
58814300
58814400
CpG site

TBX18

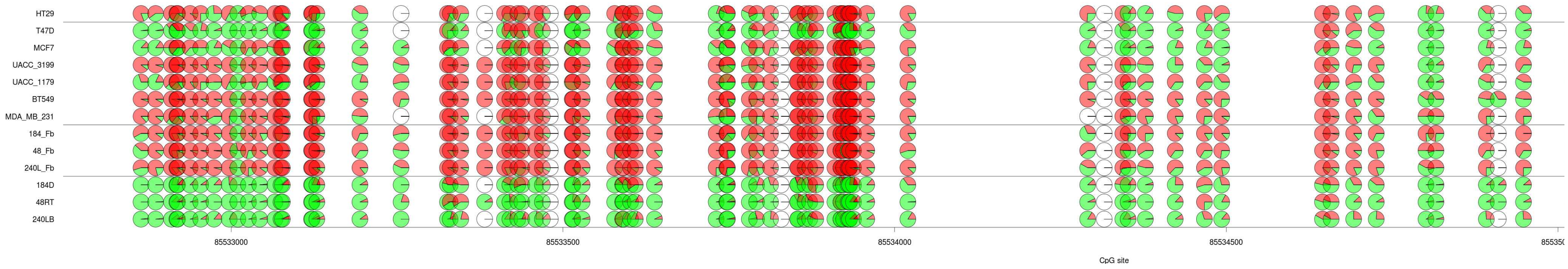
HT29
T47D
MCF7
UACC_3199
UACC_1179
BT549
MDA_MB_231
184_Fb
48_Fb
240L_Fb
184D
48RT
240LB
85533000
85533500
85534000
85534500
CpG site

TJP2

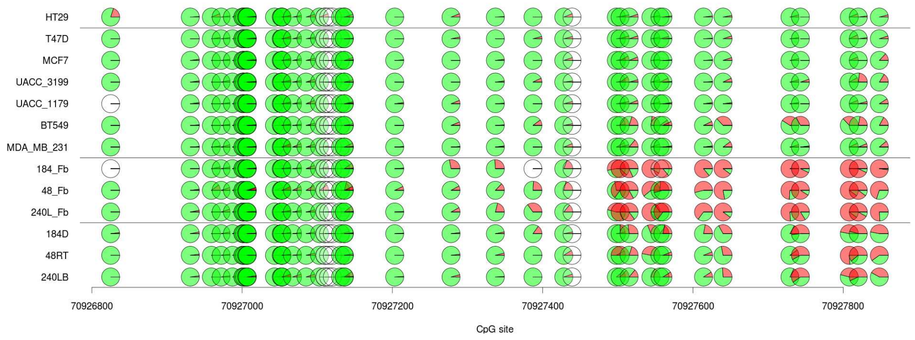
HT29
T47D
MCF7
UACC_3199
UACC_1179
BT549
MDA_MB_231
184_Fb
48_Fb
240L_Fb
184D
48RT
240LB
70926800
70927000
70927200
70927400
70927600
70927800
CpG site

TSLP

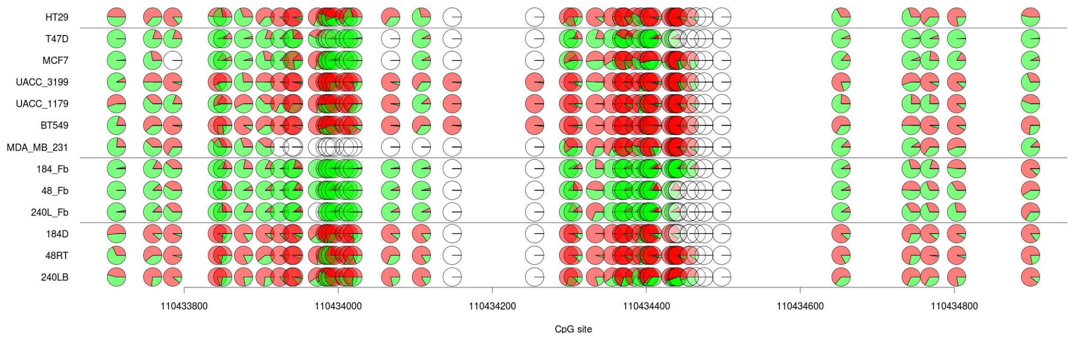
HT29
T47D
MCF7
UACC_3199
UACC_1179
BT549
MDA_MB_231
184_Fb
48_Fb
240L_Fb
184D
48RT
240LB
110433800
110434000
110434200
110434400
110434600
110434800
CpG site

TTC12

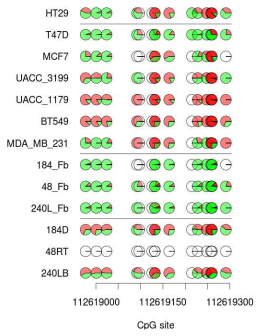
HT29
T47D
MCF7
UACC_3199
UACC_1179
BT549
MDA_MB_231
184_Fb
48_Fb
240L_Fb
184D
48RT
240LB
112619000
112619150
112619300
CpG site

ZNF295

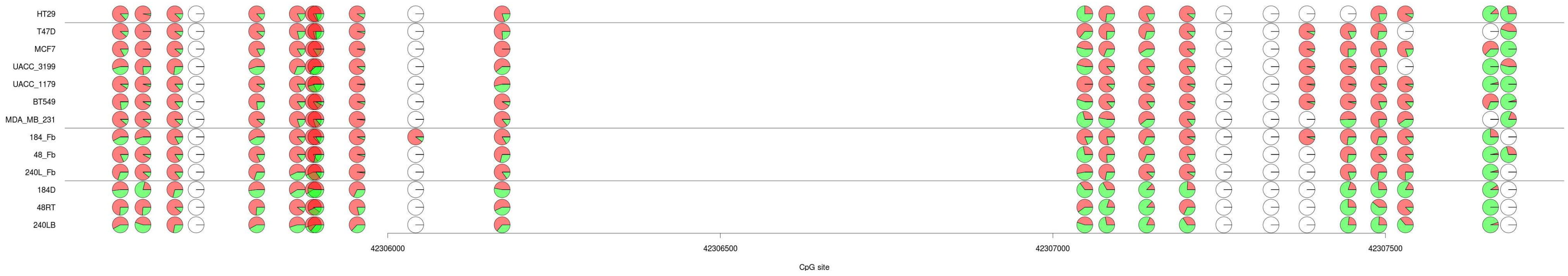

HT29
T47D
MCF7
UACC_3199
UACC_1179
BT549
MDA_MB_231
184_Fb
48_Fb
240L_Fb
184D
48RT
240LB
42306000
42306500
42307000
42307500
CpG site

ZSCAN22

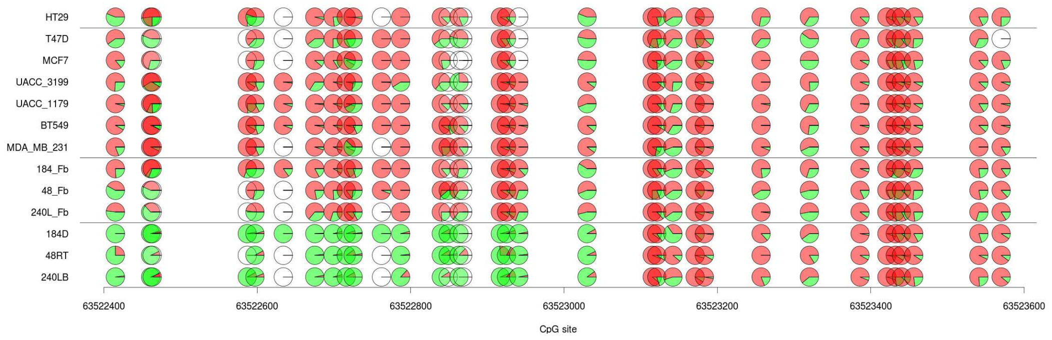
HT29
T47D
MCF7
UACC_3199
UACC_1179
BT549
MDA_MB_231
184_Fb
48_Fb
240L_Fb
184D
48RT
240LB
63522400
63522600
63522800
63523000
63523200
63523400
63523600
CpG site
